# Attenuation of 40S Ribosomal Subunit Abundance Differentially Affects Host and HCV Translation and Suppresses HCV Replication

**DOI:** 10.1371/journal.ppat.1002766

**Published:** 2012-06-28

**Authors:** Jing-Ying Huang, Wen-Chi Su, King-Song Jeng, Tien-Hsien Chang, Michael M. C. Lai

**Affiliations:** 1 Institute of Molecular Biology, Academia Sinica, Taipei, Taiwan; 2 Genomics Research Center, Academia Sinica, Taipei, Taiwan; 3 National Chen Kung University, Tainan, Taiwan; The Rockefeller University, United States of America

## Abstract

For Hepatitis C virus (HCV), initiation of translation is cap-independently mediated by its internal ribosome entry site (IRES). Unlike other IRES-containing viruses that shut off host cap-dependent translation, translation of HCV coexists with that of the host. How HCV IRES-mediated translation is regulated in the infected cells remains unclear. Here, we show that the intracellular level of 40S ribosomal subunit plays a key role in facilitating HCV translation over host translation. In a loss-of-function screen, we identified small subunit ribosomal protein 6 (*RPS6*) as an indispensable host factor for HCV propagation. Knockdown of *RPS6* selectively repressed HCV IRES-mediated translation, but not general translation. Such preferential suppression of HCV translation correlated well with the reduction of the abundance of 40S ribosomal subunit following knockdown of *RPS6* or other *RPS* genes. In contrast, reduction of the amount of ribosomal proteins of the 60S subunit did not produce similar effects. Among the components of general translation machineries, only knockdowns of *RPS* genes caused inhibitory effects on HCV translation, pointing out the unique role of 40S subunit abundance in HCV translation. This work demonstrates an unconventional notion that the translation initiation of HCV and host possess different susceptibility toward reduction of 40S ribosomal subunit, and provides a model of selective modulation of IRES-mediated translation through manipulating the level of 40S subunit.

## Author Summary

Hepatitis C virus (HCV) infection causes chronic liver diseases that threaten ∼2% of the world population. There is no effective vaccine, and the current standard therapy, the combination of interferon and ribavirin, is effective to less than 50% of genotype-1 infected patients. While antivirals targeting at specific HCV proteins might ultimately lose their effectiveness due to the emergence of resistance-associated mutations, an alternative strategy that explores the genetic stability of host factors indispensable for HCV replication may provide better therapeutic targets for anti-HCV medicine. Here, we employed a loss-of-function screen method to identify such potential targets and uncovered a potential novel anti-HCV mechanism by manipulating the biogenesis of 40S ribosomal subunit. We showed that inhibiting 40S ribosome biogenesis can selectively suppress HCV translation and thus effectively inhibit HCV replication. In contrast to the conventional thinking, the 40S ribosomal subunit can differentially affect different translational modes, and HCV translation is more sensitive to the amounts of 40S ribosomal subunit as compared to general translation in host cell. Since HCV is known to evade anti-viral effects including translational suppression elicited by interferon, our findings may help design a therapeutic strategy to supplement interferon-based therapy and minimize mutation-associated drug resistance problem.

## Introduction

Viruses lack translational apparatus, and so they rely exclusively on host machinery for their protein synthesis. Competition for components of the translational machinery between cellular mRNA and viral RNA is therefore inevitable. To gain translational advantage, viruses have evolved various strategies, among which the employment of internal ribosome entry site (IRES)-mediated initiation of translation accounts for one [1]. By adopting an initiation mechanism distinct from the predominant cellular cap-dependent initiation, differential regulation of host and viral translation is enabled, and virus translation is thus favored. For example, when cap-dependent translation is selectively repressed during picornavirus (e.g., poliovirus and enterovirus) infection, viral IRES-mediated translation prevails [2]. These viruses encode proteases capable of shutting off host translation by cleaving eukaryotic initiation factor (eIF) 4G, whose structural integrity is essential for cap-dependent, but not viral IRES-mediated, initiation of translation [3]. Although hepatitis C virus (HCV) also employs IRES-mediated initiation mechanism, no HCV protein has been reported to suppress cap-dependent translation [2]. In addition, cell death often follows the shut-off of host protein synthesis caused by virus infection [4], and yet HCV establishes chronic infection with little consequence of cytotoxicity. How HCV IRES-mediated translation is regulated in the virus-infected cells remains unclear.

HCV IRES is located at the 5′-untranslated region of HCV RNA, and is composed of highly conserved stem-loop secondary structures with specific tertiary folding [5], [6]. Skipping the requirement for eIFs in the process of directly recruiting 40S ribosomal subunit is one distinct feature of HCV IRES-mediated initiation of translation [7]. Based on the in vitro translation study using cell homogenate supernatant (Hela S10) containing complete set of translation machinery, Otto and Puglisi demonstrated that the formation of binary complex (HCV IRES and 40S ribosomal subunit) precedes the formation of 48S-like pre-initiation complex (HCV IRES, 40S subunit, eIF3 and eIF2 ternary complex) [8]. This result suggests that HCV IRES directly recruits 40S subunit and subsequently the other factors (eIF3, eIF2 ternary complex) to form 48S-like pre-initiation complex.

In contrast to the simplicity of 40S recruitment mediated by HCV IRES, cap-dependent initiation adopts a more sophisticated process, namely, it takes the coordination of various eIFs (eIF1, eIF1A, eIF2, eIF3, eIF4A, eIF4E, eIF4G) to sequentially recruit 40S ribosomal subunit to the 5′ end of capped mRNA, and then the 40S ribosomal subunit scans (energy-dependently) for the initiation codon, in a 5′ to 3′ direction [9]. The differences in the 40S ribosomal subunit recruitment process between the two distinct modes of translation initiation [7] might provide clues to the mechanism by which HCV differentially regulates host and viral translation.

Formation of a stable binary complex consisting of 40S ribosomal subunit and HCV-IRES is the very first step of HCV translation. In reconstitution experiments, the binary complex formation positively correlates with increasing concentrations of purified 40S ribosomal subunit [10]. Individual single point mutations in the IRES that compromise the binary complex forming efficiency invariably led to diminished activities of HCV IRES-mediated translation [10]. As the amount of ribosomes is largely determined by the rate of ribosomal RNA (rRNA) transcription, it is notable that HCV viral protein NS5A stimulates the transcription of rRNA by more than 10 folds [11] through hyper-phosphorylation and consequent activation of pol I DNA binding transcription factor, namely, upstream binding factor (UBF). These imply that HCV stimulates ribosome biogenesis, and thus, preferentially viral translation.

Here, in a loss-of-function screen, we have identified 40S ribosomal protein 6 (*RPS6*) as an indispensable host factor for HCV propagation in vivo. Although *RPS6* is considered a house-keeping gene, its knockdown, unexpectedly, posed little deleterious effect to the cells, while specifically reduced the HCV RNA level. We found that the abundance of 40S ribosomal subunit was attenuated by *RPS6* knockdown, which preferentially suppressed HCV IRES-mediated translation, but left global (cap-dependent) translation largely unperturbed. Knockdown of other 40S ribosomal proteins also yielded similar effects. Such selective repression of HCV translation was unique to attenuation of 40S, but not 60S, ribosomal subunit abundance. Furthermore, meta-analysis of a genome-wide screen revealed that, among the components of translation machinery, only knockdown of the 40S ribosomal subunit proteins preferentially suppressed HCV replication. These results thus suggest a distinctive role of 40S ribosomal subunit abundance in facilitating HCV translation and imply a possible role of 40S/60S ribosomal subunit ratio in differential translational regulation.

## Results

### Lentiviral vector-based RNAi screen uncovered an essential and differential role of RPS6 in HCV replication

**Figure 1:**
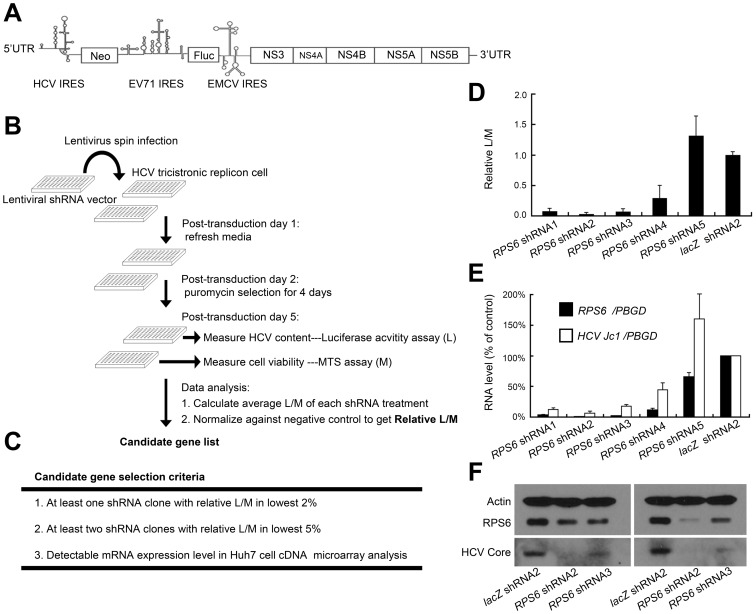
*RPS6* is an indispensible cellular factor for HCV replication as revealed by a loss-of-function screen. Fluc: firefly luciferase (A) Structural organization of the tri-cistronic HCV replicon (genotype 1). In vitro transcribed replicon RNA was electroporated into Huh7 cells and selected by neomycin to generate replicon-cell line with constitutively replicating tri-cistronic HCV. (B) Schematic diagram of the screening procedures. Lentivirus-based shRNAs were used to knock down individual host genes in replicon cells. The Lentivirus vectors bear puromycin selectable marker that allows elimination of non-transduced cells through puromycin selection. The relative L/M value serves as an indicator of HCV abundance per cell. (L, luciferase activity; M, cell viability measured by MTS assay) (C) Selection criteria for candidate genes. (D) The effects of *RPS6* shRNAs on HCV replication in the screen experiments. Significant decreases of HCV abundance per cell were shown by four of the five *RPS6* (*RPS6* shRNA1–4) knockdown. (E) The effects of *RPS6* shRNAs on the RNA levels of *RPS6* and HCV. Tri-cistronic replicon cell or HCV Jc1-infected cells were transduced with *RPS* shRNA clones. Intracellular RNAs were collected at post-transduction day 6. Quantitative RT-PCR data of HCV RNA were normalized against the level of *PBGD* RNA, an internal control. (D)(E) Error bars represent SD of averages of two independent experiments. *lacZ* shRNA was used as a negative control. (F) The correlation between *RPS6* silencing and HCV replication. HCV Jc1-infected Huh7.5 cells were transduced with shRNA-harboring lentivirus; cell lysates were harvested at post-transduction day 6 for western blot analysis using various antibodies. (anti-RPS6 antibody, Cell Signaling; anti-HCV core antibody, Thermo Scientific.)

HCV relies on host factors to complete its life cycle. To identify such cellular factors, we employed a Huh7-cell-based knockdown screening system, in which a modified tri-cistronic HCV replicon (encoding firefly luciferase) was used as a reporter (Figure 1A). In this approach, lentivirus-based shRNAs were transduced into cells for specific knockdown of the corresponding genes [12]. We simultaneously measured the luciferase activity (L) and cell viability (M) (Figure 1B). Clones that exhibited low L/M values, after normalizing against the *lac*Z shRNA treatment, presumably represented an outcome of reduction of HCV level per cell. Candidate genes were chosen on the basis of three stringent criteria (Figure 1C) for further analysis.

Out of the ∼1200 human genes screened (Table S1), *RPS6* rose up to the top of the list of candidate genes, as four of the five shRNAs targeting *RPS6* caused a significant reduction of the L/M value (>85% for shRNA1, 2, and 3) (Figure 1D).

To rule out the possibility of false-positive results arising from the tricistronic HCV replicon, which contains several non-HCV elements, the initial candidates were further validated in an infectious HCV system using Jc1 strain [13]. These four shRNAs targeting *RPS6* consistently suppressed the HCV RNA level in correlation with the decreased *RPS6* mRNA level (Figure 1E). It is interesting to note that shRNA5 inhibited *RPS6* mRNA marginally, but caused an increase in HCV RNA level. The reason for this paradoxical effect is not clear. Nevertheless, the good correlation between the *RPS6* silencing and HCV Jc1 inhibitory effects at both RNA (Figure 1E) and protein level (Figure 1F) overall suggests an indispensable role of *RPS6* in HCV replication. In addition to *RPS6*, we also identified two other hits in the same screen study, polo-like kinase 1 (*PLK1*) and proline-serine-threonine phosphatase interacting protein 2 (*PSTPIP2*). The roles of these two genes in HCV replication were reported recently [14], [15].

### Short-term depletion of RPS6 is tolerable to Huh7 cell

To investigate the mechanism of inhibition of HCV by *RPS6* knockdown, we first examined if *RPS6* knockdown caused non-specific global effects on the cells.

**Figure 2:**
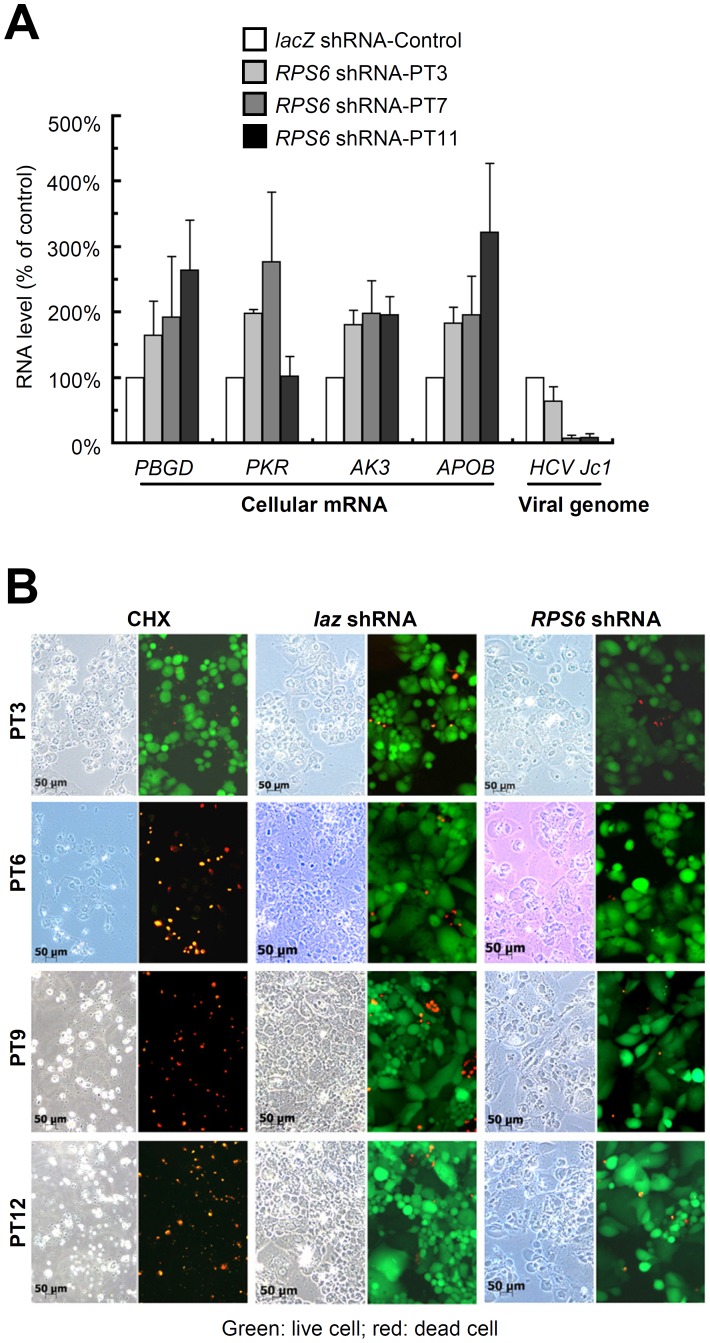
RPS6 knockdown selectively suppresses HCV replication without causing global RNA degradation or cell death. The shRNAs used were *RPS6* shRNA2 and *lacZ* shRNA2 (A) Time-course study of *RPS6* knockdown on RNA (mRNA) levels of HCV Jc1 and cellular genes. Huh7.5 cells were transduced with shRNA-expressing lentivirus and infected with HCV Jc1 two hours later. RNA was harvested at different post-transduction (PT) time points, (e.g., PT3, three days after) for quantitative RT-PCR analysis. Error bars represent SD of the averages of two independent experiments. (*PBGD*, porphobilinogen deaminase; *PKR*, protein kinase R; *AK3*, adenylate kinase 3; *APOB*, apolioprotein B). (B) Time-course cell viability evaluation of *RPS6* knockdown cells. Cell morphology is shown in bright field. Live cells with esterase activity were stained in green fluorescence, whereas dead cells with damaged plasma membrane were stained in red fluorescence. CHX, cycloheximide, a protein synthesis inhibitor used as positive control, *lacZ* shRNA, negative control.

Global RNA degradation is one possible unintended outcome that may result in decreased HCV RNA level. Such a possibility was excluded, because a time-course analysis showed that HCV RNA level was reduced by more than 80% by day 7 post-transduction, and yet the mRNA levels of four randomly selected cellular genes, including *PBGD*, *PKR*, *AK3*, and *APOB*, continued to increase (Figure 2A). This result suggests indirectly that *RPS6* knockdown specifically reduces HCV RNA synthesis, without causing global RNA degradation.

As *RPS6* encodes a component of the 40S ribosomal subunit, its knockdown may shut down global protein synthesis and cause cell death, rendering cells unable to support HCV replication. However, we found that cells expressing *RPS6* shRNA remained healthy and showed no signs of morphological alteration or deterioration of membrane integrity for at least 12 days (Figure 2B), except for a slower proliferation rate than that of the *lac*Z shRNA-treated cells (Figure S1). When cells were treated with cycloheximide (CHX), a global protein synthesis inhibitor, the entire cell population died within 6 days (Figure 2B). In contrast, the cells expressing *RPS6* shRNA or *lacZ* shRNA did not show any sign of cell death for at least 12 days post-transduction. Consistent with published evidence [16], this result demonstrate that *RPS6* knockdown did not cause cell death, at least within the 12-day period.

Another possible mechanism of inhibition of HCV replication is by confluence-associated cytostatic effect. Nelson et al. demonstrated that, masked by confluence-associated cytostatic effect, it is the shutoff of de novo nucleotide synthesis that is responsible for the inhibitory effect on HCV replication [17]. Importantly, this published study provided several critical pieces of evidence to prove that cytostatic effect is not the major cause of inhibition of HCV replication. Firstly, addition of nucleotide can recover intracellular HCV level without altering cytostatic status of the cells. Secondly, cytostatic effect alone can not reproduce confluence-associated inhibitory effect on HCV replication, as serum starvation or DNA synthesis inhibitor (aphidicolin) perturb cell cycle progression but not intracellular HCV level. These results suggest that cell growth arrest is not sufficient to account for the inhibition of HCV replication.

These results together demonstrate that the HCV inhibitory effect is not a consequence of non-specific global effects, and that *RPS6* down-regulation can be tolerated by cells without significant deleterious effects.

### *RPS6* knockdown attenuates 40S ribosomal subunit abundance without affecting polysomes

To understand the mechanism of differential effects of *RPS6* knockdown on the virus and host cells, we first examined whether 40S and 60S ribosomal subunits and polysomes were differentially affected by *RPS6* knockdown. Polysome profile analysis was performed and each ribosome species was quantified by integrating the areas under each peak.

**Figure 3:**
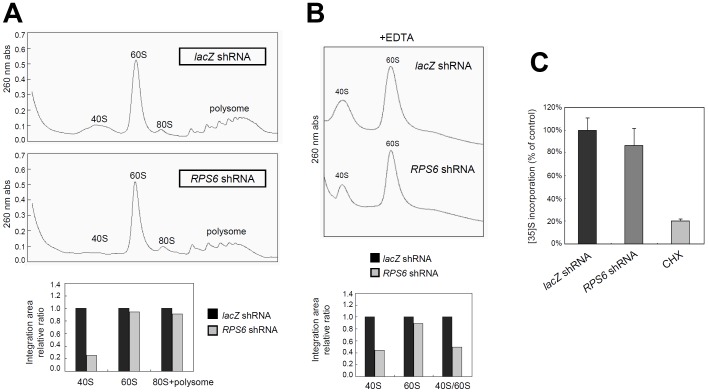
*RPS6* knockdown reduces free 40S ribosomal subunit but has no effects on general translation. The shRNAs used here were *RPS6* shRNA2 and *lacZ* shRNA2. (A)(B)(C) Huh7.5 cells transduced with *RPS6* shRNA vector or *lacZ* were harvested at post-transduction day 5. Polysome profile analysis of *RPS6* knockdown cells in the absence (A) or in the presence (B) of EDTA. Quantification of each peak area is shown in the lower panel. (C) The effect of *RPS6* knockdown on L-[^35^S]-methionine incorporation. Huh7.5 cells (1×10^6^) were pulse-labeled with L-[^35^S]-methionine for 15 min and the tricholoroacetic-acid precipitates from cell extracts were measured for [^35^S]-incorporation. CHX, cycloheximide. Error bar, SD of independent triplicates.

The amount of free 40S ribosomal subunit was significantly decreased in the *RPS6*-knockdown cells as compared to the control *lacZ* shRNA-transduced cells; in contrast, the amounts of 60S, 80S and polysomes were not affected by *RPS6* knockdown (Figure 3A). These results suggest that when *RPS6* was knocked down, ribosomes that are actively engaged in translation (shown as 80S and polysomes), stayed relatively unchanged, and only free 40S subunits, which are not involved in translation (shown as the 40S peak), was preferentially affected.

In the presence of EDTA, which dissociates ribosomes into 40S and 60S subunits, the total amount of 40S ribosomal subunit was reduced by 50% in the *RPS6*-knockdown cells. These 40S ribosomes were most likely derived from the 80S ribosomes and polysomes; these results suggest that there were still a substaintial amount of 40S ribosome in the polysomes, even though there was little free 40S ribosome in the *RPS6*-knockdown cells. In contrast, the 60S ribosome peak remained relatively unchanged in the *RPS6*-knockdown cells, as compared to that in *lac*Z shRNA-treated cells (Figure 3B). These results suggested that the knockdown of RPS6 affected primarily the amount of free 40S ribosome while leaving the polysomes essentially intact. These data are highly reproducible. These results also suggest that overall translation in the RPS6 knockdown cells should not be affected.

Consistent with the polysome profile analysis, L-[^35^S]-methionine incorporation study showed that knockdown of *RPS6* did not significantly reduce global protein synthesis (Figure 3C). As a control, cycloheximide treatment reduced protein synthesis by more than 80%. These data together demonstrate that despite the reduction of 40S ribosome abundance following *RPS6* knockdown, the overall translation in the cells was not significantly affected.

### Translation mediated by HCV IRES is preferentially suppressed in *RPS6* knockdown cells

**Figure 4:**
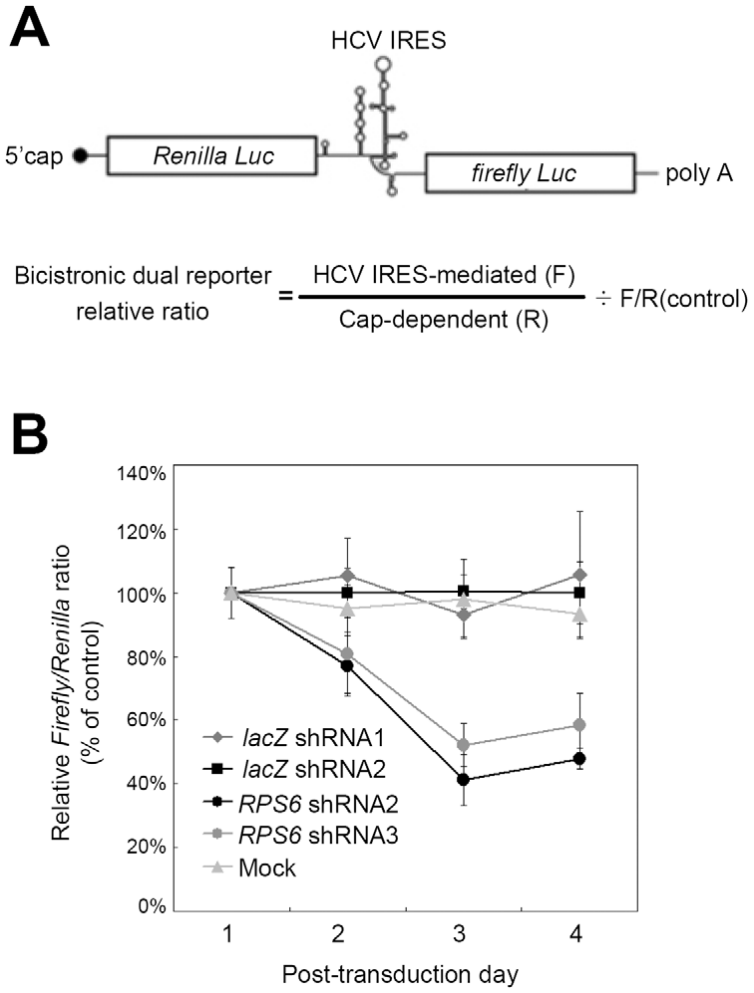
*RPS6* knockdown preferentially suppresses HCV IRES-mediated translation over cap-dependent translation. (A) Schematic illustration of the bicistronic dual reporter. The translation of Renilla luciferase ORF is driven by cap-dependent translation initiation, while that of firefly luciferase ORF is mediated by HCV-IRES. (B) Time-course study of *RPS6* knockdown on bicistronic dual reporter ratio. Huh7.5 cells were first transduced with lentivirus-based shRNA, and then transfected with bicistronic dual reporter DNA at different post-transduction time points. Dual reporter activities were assayed 20 hours post-transfection. *lacZ* shRNA, negative control. Error bars represent SD of 4 replicates.

Different from the general translation of the cell, the majority of which utilizes cap-dependent initiation of translation, HCV translation relies on its IRES-mediated initiation, where direct binding of eIF-free 40S ribosome to IRES is the crucial first step [8]. To test whether cap-dependent translation and IRES-dependent translation exhibit differential sensitivity toward *RPS6* knockdown, we employed a standard bicistronic reporter assay, in which an HCV IRES element was inserted between *Renilla* and firefly luciferase ORFs (Figure 4A). We found that the relative ratio of the two luciferase activities dropped over time to about 50% after transduction with each one of the two different shRNAs of *RPS6* (Figure 4B). In contrast, *lac*Z shRNAs did not alter the ratio (Figure 4B). These results showed that RPS6 depletion selectively suppressed HCV IRES-mediated translation.

### Knockdown of other *RPS, but not RPL*, also inhibits HCV IRES-mediated translation and HCV replication

**Figure 5:**
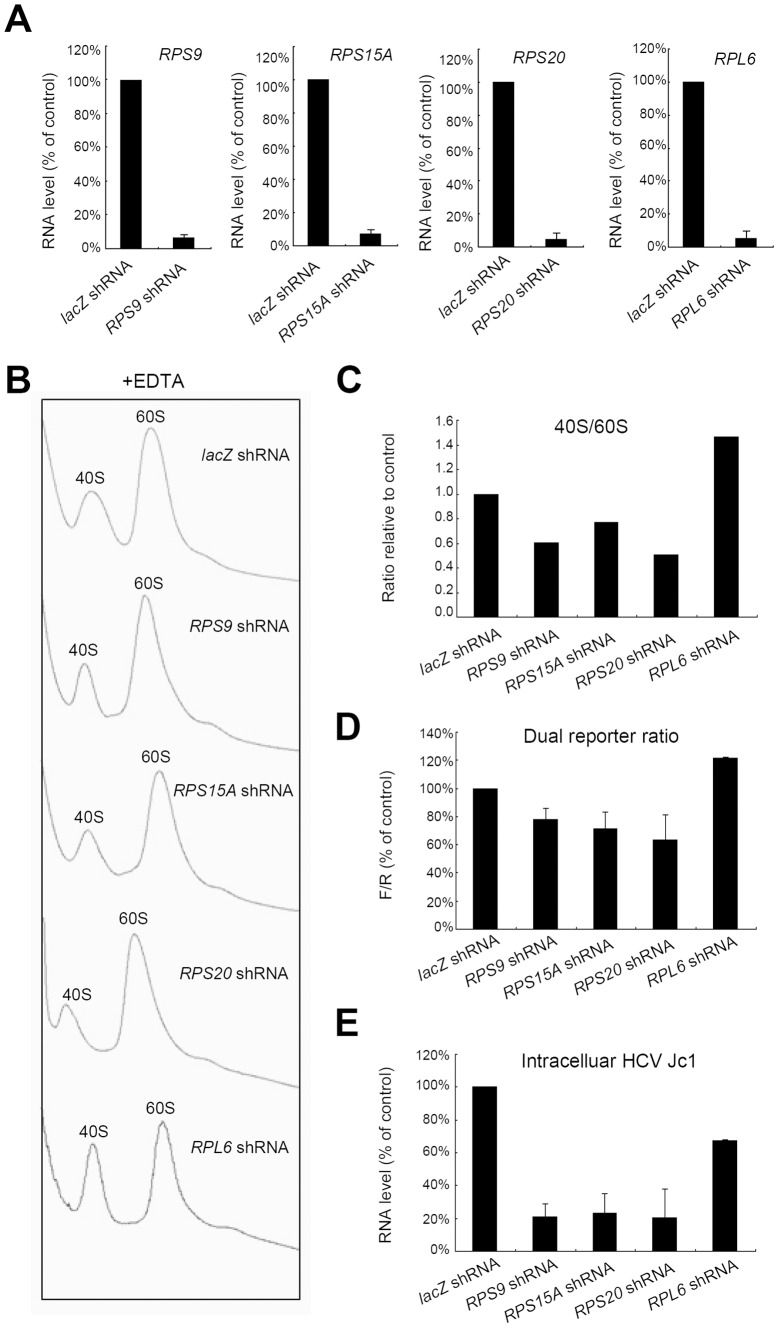
HCV suppressive effects caused by knockdown of other ribosomal proteins. (A) The knockdown efficiency of shRNAs targeting *RPS9, RPS15A, RPS20* and *RPL6*. Huh7.5 cells were transduced with the various lentivirus-based shRNA. RNA was extracted for quantitative RT-PCR analysis at post-transduction day 6. (*RPS*, small subunit ribosomal protein; *RPL*, large subunit ribosomal protein). (B) The effect of *RPS* and *RPL* knockdown on the total amounts of individual ribosomal subunits. Cytosols of Huh7.5 cells transduced with lentivirus-based shRNA were collected at post-transduction day 5 for ribosome profile analysis in the presence of EDTA. The rRNA was detected by 260 nm absorbance peaks. (C) The effect of *RPS* and *RPL* knockdown on the 40S/60S ratio. 40S/60S ratios were calculated from the quantification of integration areas shown in panel (B). That for the *lacZ* shRNA control is set at 1. (D) The effect of *RPS* and *RPL* knockdown on bicistronic dual reporter ratio. Huh7 cells were transfected with bicistroinic dual reporter pDNA at post-transduction day 4. Reporter activities were assayed 20 hours post-tranfection. The ratio in the *lacZ* shRNA control is set at 100%. (E) The effect of *RPS* and *RPL* knockdown on the RNA level of infectious HCV. Huh7 cells infected with HCV Jc1 two hours post-transduction. Intracellular total RNA was extracted for RT-qPCR analysis at post-transduction day 6. (A)(D)(E) Error bar, SD of independent replicates. The RNA level in the *lacZ* shRNA cells is set at 100%.

To test whether inhibition of HCV IRES-mediated translation is a general property of the reduction of 40S subunit abundance, or a unique property of RPS6, we examined the effects of knockdown of other small subunit ribosomal protein *(RPS)* genes. The shRNAs targeting *RPS9*, *RPS15A* and *RPS20* efficiently reduced target gene mRNA expression level by more than 90% (Figure 5A); knockdown of these *RPS* genes specifically caused reduction of the ratio of the 40S/60S ribosomal subunit by 20–50% (Figures 5B and 5C) (Figure S2 and S3). Correspondingly, the relative ratio of the luciferase activity of the dual reporter RNA, representing IRES-dependent vs. cap-dependent translation, also dropped by 20∼40% following the knockdown of these *RPS* (Figure 5D). Furthermore, there was an 80% reduction of HCV Jc1 RNA level in the cells expressing shRNA targeting these *RPS* genes (Figure 5E). The finding that HCV RNA level was reduced by a higher extent (80%) than was the translation activity (20–50%) can be explained by the fact that the HCV RNA level reflects the cumulative effects of translation and replication, the latter of which is also affected by the amount of viral polymerase protein. Therefore, through the cumulative effects, the suppression of HCV IRES activity would result in magnified inhibitory effect on HCV replication (Figure 5).

These results demonstrated that individual *RPS* knockdown caused suppression of HCV IRES-mediated translation, and thus lowered HCV replication efficiency. Also, they suggest that the HCV inhibitory effect is most likely due to the reduction of the amount of 40S ribosomal subunit.

### HCV inhibitory effect is specific to 40S ribosome attenuation rather than general perturbation of components of translation machinery

To determine if such preferential suppression of HCV is specific to the attenuation of 40S ribosomal subunit or is due to perturbation of the translation machinery in general, we tested the effect of 60S attenuation on HCV replication. Knockdown of large ribosomal protein 6 (*RPL6*) caused a more than 90% reduction of *RPL6* mRNA amount (Figure 5A) and a correspondingly reduced amount of 60S ribosome (Figure 5B) (∼40% increase of the 40S/60S ratio, Figure 5C). However, unlike the *RPS* gene knockdown, the *RPL6* knockdown did not alter the dual reporter ratio (Figure 5D); also, it suppressed HCV RNA level only slightly (Figure 5E). These results suggest that the suppression of HCV IRES-mediated translation is specific to the attenuation of the abundance of 40S subunit.

**Figure 6:**
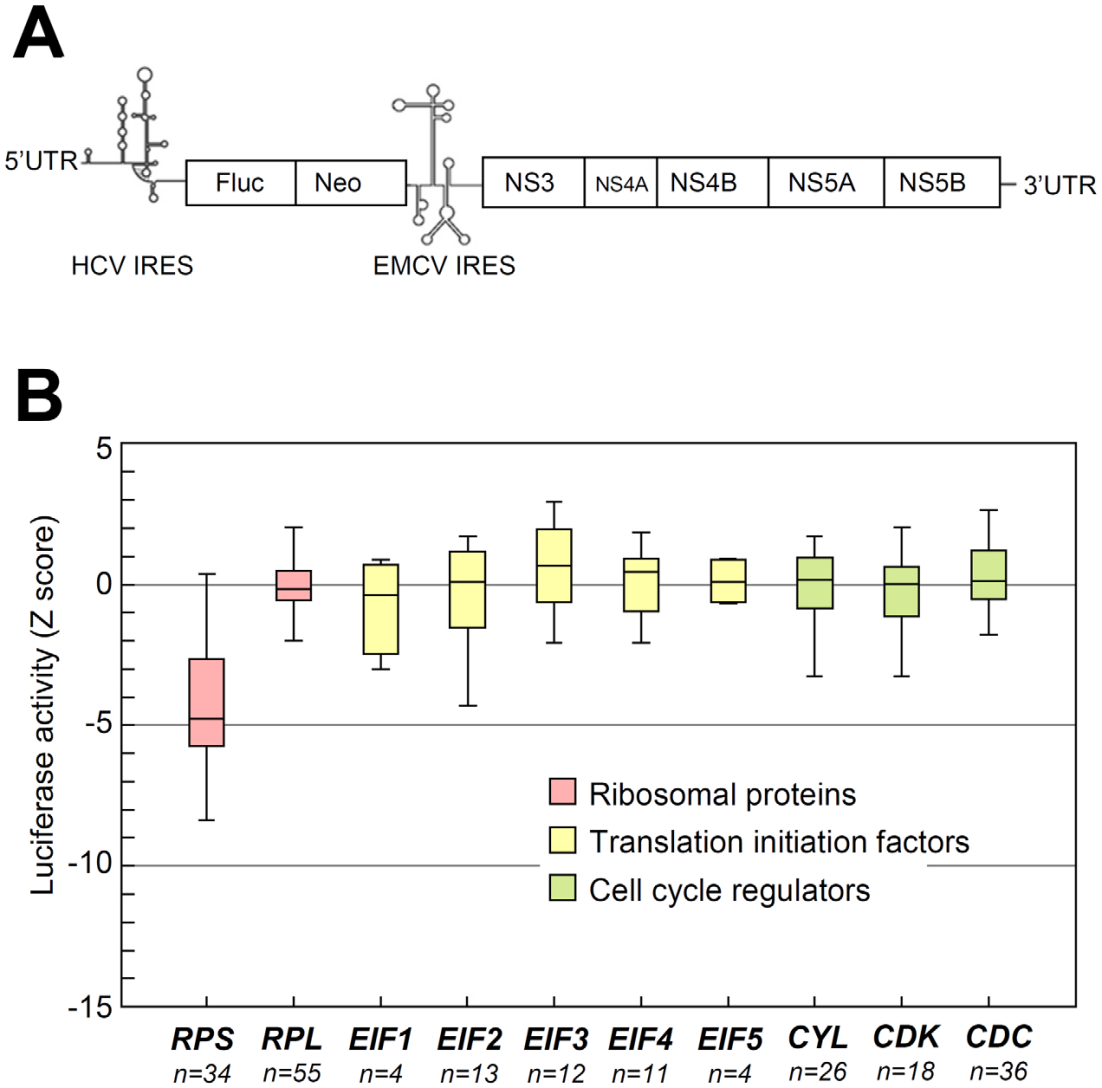
Meta-analysis of the roles of various translation related genes in a genome-wide screen. (A) Schematic illustration of the bicistronic HCV subgenomic replicon used in the published genome-wide screen experiment [19]. (B) Meta-analysis of the genome-wide screen experiment. Genes that are involved in the translation and cell-cycle regulation are selected for meta-analysis. Those genes with closely-related functions were grouped together for box plot (indicated by different colors). The screen result is shown as Z score, which is the number of standard deviations of the experimental luciferase activity above the median plate value. Negative Z scores indicate inhibition of HCV replication.

Other than ribosomal subunits, the translation machinery also contains components such as translation initiation factors, which are involved in the regulation of initiation of cap-dependent translation [18]. To determine whether knockdown of these components would result in inhibitory effect on HCV replication, we performed a meta-analysis of a genome-wide screen result based on a published report [19]. This genome-wide screen used siRNA library to monitor the effects of knockdown of cellular genes on the firefly luciferase activity that reflects replication efficiency of a bicistronic subgenomic HCV replicon (Figure 6A). The result of analysis is shown as Z score, which is the number of standard deviations of the experimental luciferase activity above the median plate value in the genome-wide screen. Negative Z scores indicate inhibition of HCV replication. The meta-analysis (box plot) (Figure 6B) reveals that, among all components of translation machinery, only those that disturb the expression of 40S ribosomal proteins (*RPS*, 34 genes) consistently exhibited significant HCV inhibitory effect (median Z score around -5). In contrast, knockdown of the 60S ribosomal proteins (*RPL*, 55 genes), components of eukaryotic translation initiation factors (*eIF1* to *eIF5*, 44 genes in total), cell cycle regulation-related genes such as cyclins (*CYL*, 26 genes), cyclin-dependent kinase (*CDK*, 18 genes) and cell division cycle-related genes (*CDC*, 36 genes), did not preferentially inhibit HCV replication (median Z score around 0) (Figure 6B). These results suggest that the components of 40S ribosomal subunit are distinct from any other components of the translation machinery in playing key roles in HCV replication. It is noteworthy that the result of the knockdown of cell cycle-related genes is consistent with our conclusion (Figure 2) that decrease of cell proliferation rate does not lead to preferential suppression of HCV replication.

It has not escaped our notice that the genetic structure of the bicistronic HCV replicon used in the screening of the cellular genes [19] for this meta-analysis might have caused overestimation of the effects on viral replication, because silencing of *RPS* genes would also directly affect the expression of luciferase reporter, whose translation is driven by HCV IRES. The same reservation applies to the constructs used in our study. Nonetheless, the possibility of overestimation in our case was minimal, since our shRNA treatments were verified by direct biochemical evidence that silencing of small ribosomal protein genes significantly inhibited the replication of infectious HCV Jc1 by more than 70% (Figure 5E).

In summary, our results show that *RPS6* knockdown attenuates the abundance of 40S ribosomal subunit, which, in turn, preferentially suppresses HCV IRES-mediated translation, and thus inhibits HCV replication without perturbing general translation and cell survival. Importantly, such selective HCV inhibitory effect is specific to the attenuation of 40S, but not that of other components of the translation machinery.

## Discussion

The findings reported here support an unconventional concept that 40S ribosomal subunits can differentially affect different modes of translation. The translation efficiency of HCV, but not that of the cell, is highly correlated with the abundance of 40S ribosomal subunit. Reduction of 40S subunit after knockdown of the various 40S ribosomal proteins *(RPS)*, consistently suppressed the translational activity mediated by HCV IRES (Figures 5B, 5C and 5D), and led to decrease of HCV RNA level (Figure 5E). In contrast, cellular general translation, where cap-dependent initiation predominates, remained largely unperturbed despite the near 50% reduction of total 40S subunit (Figure 3B), as demonstrated by polysome profile analysis (Figure 3A) and S[35]-methionine incorporation study (Figure 3C). Furthermore, the reduction of 60S ribosomal protein (*RPL*) (Figure 5) or other translation initiation factors (*eIF*) did not have such an effect (Figure 6). Taken together, these results showed that the reduction of 40S subunit differentially affected the two modes of translation initiation adopted by HCV and host cell respectively, indicating a unique role of 40S ribosomal subunit in translational regulation.

Although the reduction of HCV translation by *RPS* gene knockdown was only modest (approximately 20–50%), the reduction of HCV RNA level was substantial (75%) (Figure 5E). This is most likely due to the fact that the reduction of viral protein synthesis (e.g. RNA polymerase) also led to reduction of viral RNA replication, which determines the amount of positive strand HCV RNA available for translation, thus amplifying the suppressive effects. In addition to the cumulative effect originating from translation-replication coupling, the stability of HCV RNA might be another contributing factor. It is likely that when HCV RNA cannot be efficiently translated or replicated, it is rapidly degraded. This possibility will need to be verified by future experiments.

Several possibilities may explain the general increase of cellular mRNA in *RPS6* knockdown cells (Figure 2A). One explanation is that the cellular energy originally devoted to ribosome biogenesis, which accounts for ∼80% [20] of cellular energy consumption, has been significantly reduced in *RPS6* knockdown cells, and the saved energy was then shifted to other energy-competing cellular processes, such as mRNA transcription. The other possibility is that it might reflect an adaptive feedback to maintain homeostatic gene expression level in response to the slightly decreased translation efficiency caused by the reduction of 40S ribosomal subunit. The cellular mRNA levels were elevated to compensate for the reduction of protein expression level.

It has been shown that quantitative reduction of *RPS* leads to defects at various stages of 18S rRNA maturation [21], [22], causing accumulation of precursor rRNA (45S) and reduction of mature 18S rRNA. The reduction of both *RPS* and 18S rRNA will lead to decreased level of 40S ribosomal subunits. Our study showed that, in *RPS* knockdown cells, the total amount of 40S ribosomal subunit was reduced by various degrees (Figures 3B and 5B), whereas that of 60S ribosomal subunit remained unaffected. These results suggest that, despite the fact that 40S and 60S subunits share the same rRNA precursor, selective reduction of 40S ribosomal subunit is achievable through reducing its corresponding ribosomal proteins. Knockdown of each one of the *RPS* proteins studied in this report resulted in the reduction of 40S ribosomal subunit. However, we could not rule out the possibility that these RPS proteins may also affect certain specific functions of ribosome. It has been reported that knockdown of *RPS25* does not affect the level of 40S ribosomal subunit, but affects HCV translation [23]. Thus, RPS25 probably affects structure of 40S ribosome; in either case, the abundance and structural integrity of 40S ribosome play key roles in differentiating HCV and host translation.

Unlike the traditional view that IRES-containing viruses are particularly sensitive to the inhibition of general translation [24], our findings indicate that, for HCV, the effect of 40S attenuation is different from that of 60S attenuation or perturbations of translation initiation factors, and suggest a distinction between HCV and other IRES-containing viruses. The key difference between HCV IRES-mediated translation and cap-dependent or other viral IRES-mediated translation lies in the steps of 40S subunit recruitment that use different forms of 40S subunit [7]. Based on the in vitro reconstitution assays, it has been shown that eIFs are not required for the initial binding of 40S subunits to the HCV IRES [8], [10], whereas most eIFs are utilized in those of cap-dependent and encephalomyocarditis virus (EMCV) IRES-mediated translation [7]. For the latter cases, 43S pre-initiation complex (consisting of 40S subunit, eIF1, eIF2, eIF3, initiator tRNA and GTP [18]) is involved in 40S ribosomal subunit recruitment, and the eIFs (eIF4A, eIF4B) are indispensible in facilitating the migration of 40S subunit to the translation initiation sites. However, the in vivo evidence for this model is still not available. These differences may explain the differential sensitivity of HCV translation to the amounts of 40S ribosome.

In *RPS6* knockdown cells, the near 50% reduction of total 40S ribosomal subunit (Figure 3B), affected only the amount of free 40S subunit (the 40S that are not engaged in translation) but not that of 60S, 80S and polysomes (Figure 3A). It is likely that 40S ribosomal subunits are recycled from polysomes immediately after translation termination. Recycling of ribosome after translation termination is facilitated by the binding of eIF1 and eIF3 to 40S ribosomal subunit [25]; once eIF2-GTP-Met-tRNA^Met^ joins, this form of 40S subunit readily forms 43S pre-initiaition complex [9], and thus supports cap-dependent translation. Therefore, when the levels of 40S ribosomal subunits were partially depleted after *RPS6* knockdown (Figure 3B), cap-dependent translation was maintained (Figure 3C), presumably by the recycled ribosome, while that of HCV IRES was preferentially suppressed (Figure 4B), very likely due to the shortage of eIF-free 40S subunit.

In all the polysome profiling experiments presented here (Figures 3A, 3B and Figure 5B), each treatment was performed using 10 (6-well-) plates, which were pooled together for polysome analysis. The boundaries used for quantifying peaks are shown in Figure S4. Our results are consistent with published evidence [16], [21], [22] demonstrating that silencing of ribosomal protein reduces the abundance of the corresponding ribosomal subunit.

**Figure 7:**
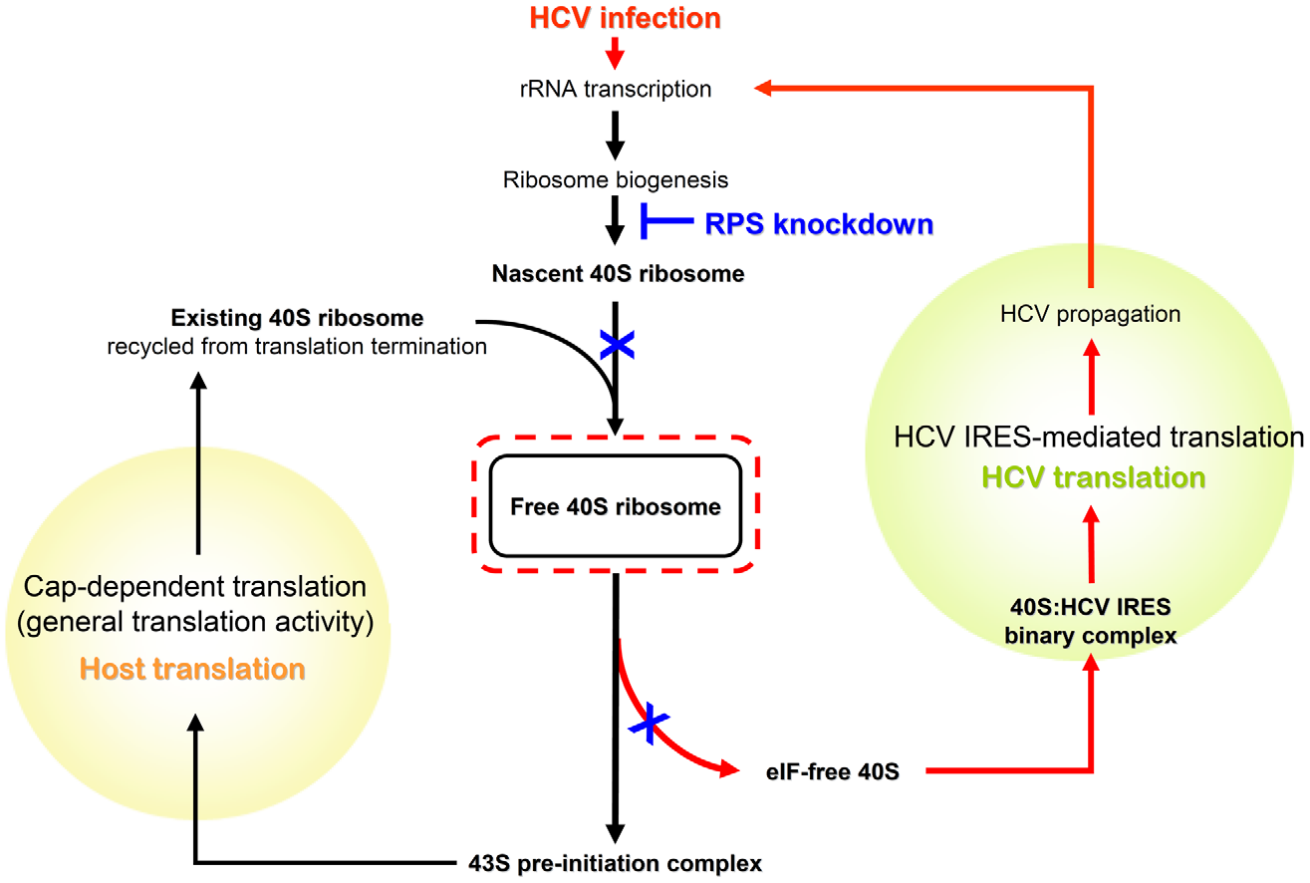
The abundance of free 40S ribosomal subunit differentially affects the translations of host and HCV. The initiation of cap-dependent and HCV IRES-mediated translation both require free 40S subunit, however, in different forms; namely, 43S pre-initiation complex (eIF-bound form) for the former [18], and eIF-free 40S subunit for the latter [8], [10], respectively. The amount of 40S ribosomal subunit is boosted by HCV infection [11] and attenuated by *RPS* knockdown. During HCV infection, ribosome synthesis was enhanced [11]; as a result, the level of eIF-free 40S subunit increases as the number of free 40S exceeds that of available eIF. Consequently, elevation of eIF-free 40S subunit facilitates HCV IRES-mediated translation, and explains how HCV gains translational advantage through stimulating ribosome biogenesis [11]. In contrast, when free 40S is reduced by *RPS* knockdown, recycled 40S subunits become the main source for new initiation events. Ribosome recycling after translation termination is facilitated by eIF1 and eIF3 [25]. Those recycled 40S subunits are in favor of the formation of 43S pre-initiation complex, therefore, cap-dependent translation is sustained, but HCV IRES-mediated translation is preferentially suppressed in response to the reduction of total 40S ribosome.

Together with published data, we propose a model of how the translational advantage of HCV varies in response to the rises and falls of 40S subunit abundance, as illustrated in Figure 7. The amount of free 40S ribosomal subunit fluctuates in response to the total amount of 40S ribosomal subunit, whose biogenesis is boosted by HCV infection [11] and attenuated by *RPS* knockdown. Thus, during HCV infection, ribosome synthesis increases [11]; as a result, the level of free 40S subunit increases and so does that of the eIF-free 40S subunit, which then facilitates HCV IRES-mediated translation. On the other hand, as *RPS* knockdown blocks the maturation of nascent 40S ribosomal subunits, the reduction of total 40S subunit then selectively reduces availability of free 40S ribosomal subunit. Accordingly, 40S subunit recycled from translation termination sustains the formation of 43S pre-initiation complex [25] but not that of eIF-free 40S; as a result, cap-dependent translation remains unaffected, resulting in preferential suppression of HCV IRES-mediated translation.

The conclusion that the availability of the 40S ribosomal subunit is crucial for HCV proliferation is compatible with the result that depletion of RPL6 increased translation of the HCV IRES-driven firefly luciferase in the bicistronic reporter (Figure 5D). Since the ratio of 40S∶60S subunits was increased (Figure 5C) in cells depleted of RPL6, more free 40S ribosomal subunit would become available for forming a complex with the HCV RNA and thus compete with cellular mRNAs for available 60S ribosomal subunits. Interestingly, the increase in HCV translation nonetheless suppressed the HCV RNA levels in Jc1-infected cells to 70% of the control. According to published data [22], silencing of *RPS* only had slight effects on cellular polysomes, whereas silencing of *RPL* significantly decreased total amounts of polysomes. Since general translation is affected substantially by knockdown of *RPL*, it is conceivable that, in *RPL* knockdown cells, the decrease of overall translation activity ultimately will negatively affect the production of HCV viral proteins, despite the elevated 40S/60S ratio favoring HCV IRES-mediated translation.

There are growing lines of evidence showing that ribosomal protein may be highly regulated to exert specific translational control in gene expression [26], which leads to a provocative model of “ribosome code” [27]. Consistent with this concept, our data provide a model that alteration in ribosomal subunit 40S/60S ratio can differentially affect cap-dependent and HCV IRES-mediated translation, pointing out a possible mechanism that ribosomal proteins exert specific control through fine tuning the ratio of 40S/60S ribosomal subunit.

In this loss-of-function screen study, our tricistronic replicon was originally designed for probing host factors involved in HCV replication in general, not limited to the translation of HCV (here the HCV IRES drives translation of the neo gene (Figure 1A), which is irrelevant in the time frame of the screen experiment). Nonetheless, this system can still identify essential factors required by HCV IRES-mediated translation for two main reasons. One is that all these viral IRES elements that drive the expression of reporter and HCV polyprotein respectively may share the same cellular factors [24]. The candidates that are not caused by the effects on HCV IRES would be excluded by the confirmation study using infectious HCV. The other reason is that the 5′UTR region of HCV harbors not only IRES crucial for translation, but also the sequence crucial for genome replication [28]; therefore, due to tight coupling of translation and replication of IRES-containing virus [29], [30], [31], including HCV [32], many host factors, such as La autoantigen [33], [34], *PTB* [35], and *PCBP2* [36], are involved in both functions. *RPS6* was selected under such a condition.

Current therapy against HCV infection relies mainly on interferon (IFN)-based regimens [37], based on the anti-viral defenses activated by IFN signaling [38]. However, HCV has evolved various strategies to evade host defense, such as blocking IFN-mediated antiviral defense by perturbing the production and signaling of IFN [39]. Moreover, some HCV genotypes are intrinsically resistant to IFN [40]. Besides, the current standard therapy is only effective for <50% of genotype-1- and ∼80% of genotypes 2 and 3-infected patients [41]. Although novel therapies developed to target HCV viral proteins are promising, they may eventually be compromised by rapid emergence of resistance-associated mutations of HCV [42]. Here, our findings provide a new anti-HCV strategy by manipulating host factors, namely, the level of 40S ribosomal subunit, to act against the translational advantage of HCV. This strategy not only inhibits viral propagation effectively, but also provides a solution to current mutation-associated drug resistance problems [43].

## Materials and Methods

### Cells

Cells (Huh-7.5, HCV-tricistronic replicon cell, and 293T) were cultured in Dulbecco's modified Eagle's medium (DMEM) supplemented with 10% fetal bovine serum, nonessential amino acids, 100 units/ml penicillin, and 100 µg/ml streptomycin at 37°C in a CO_2_ (5%) incubator. To ensure cell line consistency, no more than 15 passages of subculture were employed.

### Generation of HCV-EV71I-Luc replicon cells for shRNA screen

The HCV tricistronic replicon used in this study was modified from the HCV-1b-neo45 replicon construct [44] by inserting an EV71-IRES-driven firefly luciferase ORF between Neo ORF and the EMCV IRES. The tricistronic replicon RNA was produced by in vitro transcription, and electroporated into Huh-7 cells (ECM630 Electroporator, BTX Harvard Apparatus; 975 µF and 220 V) and selected with G418. The colony with the highest luciferase activity and the highest HCV protein level was chosen and maintained in G418 (0.5 mg/ml)-containing medium for subsequent experiments.

### Lentivirus production and titer determination

All plasmids for lentivirus production were provided by the National RNAi Core Facility, Academia Sinica, Taiwan. For lentiviral production, pCMV-ΔR8.91 (for packaging), pMD.G (for envelope proteins), and an individual shRNA construct were transfected into 293 T cells using Trans-IT (Mirus Bio) (see Protocol S1). The viral titer was determined in Huh-7 cells by using a cell viability assay (i.e., the Relative Infectious Unit [RIU] method) (see Protocol S2).

### High-throughput shRNA screen

The HCV-EV71-Luc-replicon-containing cells were plated in 96-well plates (1×10^4^ cells per well) 24 h prior to transduction. Cells were transduced with lentiviruses at a multiplicity of infection (MOI) of ∼3 in the presence of Polybrene (hexadimethrine bromide; 8 µg/ml) by spin infection (1,100×*g*, 15 min, 37°C) and incubated at 37°C for 24 h. The cells were incubated with fresh medium for another 24-h incubation and finally incubated in the media containing puromycin (2 µg/ml). Luciferase activity (L) and cell viability (M) were measured 5 days after the lentiviral transduction. L was determined by using a Bright-Glo Luciferase Assay System (Promega) and M was measured by using an MTS assay (CellTiter 96 Aqueous Non-Radioactive Cell Proliferation Assay, Promega). The relative L/M ratios, which were normalized against the L/M value of *lac*Z-shRNA-transduced control cells, were used to evaluate the knockdown effects on HCV replication.

### Lentivirus infection/shRNA transduction

All lentivirus-based shRNA knockdown experiments were done at MOI = 2 to ensure high transduction efficiency and uniform utilization of the microRNA processing machinery in the cells. Transduction and puromycin selection were done as described above. For the shRNA sequences used in this study, please refer to Table S2.

### HCV infection system

The pJc1 plasmid, which contains a chimera genome of HCV J6CF/JFH1, was constructed as previously described [13]. Full-length JC1 genomic RNA was produced by in vitro transcription of pJc1, and electroporated into Huh7.5 cells, and incubated for 24 hrs. Jc1 viral particles were then collected from the cell culture supernatant at 3 day after transfection for further expansion. Viral particles were titered by counting the number of infected cell colonies by immunostaining HCV core protein as described [45]. For studying the effects of shRNA on Jc1 infection, Huh7.5 cells were first transduced with lentivirus-based shRNA for 2 h and then incubated with Jc1 virus suspension (MOI = 1) for 1 h at 37°C. The intracellular virus RNA was determined at various time points after infection.

### Quantitative detection of HCV and cellular mRNAs

Total RNAs were extracted from cells by using RNeasy Mini Kit (Qiagen) and converted into cDNAs using SuperScript III First-Strand Synthesis System (Invitrogen). The primers for reverse transcription are oligo(dT)_20_ and an HCV-specific primer (5′-CACTCGCAAGCACCCTATCA-3′). For real-time PCR analysis, we followed the standard TaqMan strategy using the Universal Probe Library and the LightCycler 480 Real-Time PCR System (Roche Diagnostics) (Table S3). Each quantitative PCR reaction was performed in duplicates. Data were normalized against the quantity of *PBGD*, which served as an internal control.

### Cell viability assay

Cells were stained based on membrane integrity and intracellular esterase activity by using LIVE/DEAD Viability/Cytotoxicity Kit for mammalian cells (Invitrogen). Cells seeded in 12-well plates were washed with 1× PBS two times to remove serum esterase in the medium, and followed by incubation with the staining reagent (2 µM Calcein AM, 4 µM Ethidium homodimer in 1× PBS) at room temperature for 30 min. Images of stained cells were acquired by using a Zeiss inverted fluorescent microscope system (Axio Observer A1).

### Polysome profile analysis

Cell lysate was prepared by mixing 2.5×10^6^ cells with 1-ml RNase-free lysis buffer (0.5% Nonidet P-40, 500 U/ml RNAse inhibitor (Invitrogen), 1 mM PMSF, 20 mM DTT, 150 µg/ml cycloheximide) on ice and centrifuged at 12,000×g for 5 min at 4°C to remove nuclei. The RNA concentration in the supernatant was then determined. Linear sucrose gradient (11 ml) was prepared by mixing equal volume of 10% and 60% sucrose stock solutions (in 300 mM KCl, 5 mM MgCl_2_, 10 mM HEPES [pH 7.4]) using Gradient Master (Bio-Comp). An equivalent of 300-µg RNA of lysate was loaded onto a sucrose gradient, which was then centrifuged in a SW41 rotor (Beckman) at 21,000×g for 2 h at 4°C. We used the Density Gradient Fractionation System (ISCO) to fractionate the gradients at a flow rate of 0.75 ml/min with the UV-detector sensitivity set at 1.0. To dissociate ribosomes into subunits for similar analysis, EDTA was included in the lysis buffer (100 mM) and sucrose gradient buffer (25 mM).

For quantitative analyses of polysome profile, the area of integration of each peak was defined as the area enclosed by the trace lines and the boundary lines. The boundary line is defined by two points at which the slope of the trace line exhibits the biggest difference with that of the adjacent point (See Figure S3 and S4). Paper boards were cut along the contour of these integration areas and weighed; the weights of these paper boards were proportional to the sizes of the integration areas and used for quantitative analyses.

### Measuring protein synthesis by L-[^35^S]-methionine incorporation

Lentivirus-transduced cells (1×10^6^) were first incubated in methionine-free medium for 1 h at 37°C. The medium was then replaced with the same media containing 0.2 mCi of L-[^35^S]-methionine (>1000 Ci/mmol; NEG709A005MC, PerkinElmer) in 1-ml volume and further incubated for 15 min. Cells were then washed with PBS and resuspended in 100 µl of BSA (1 mg/ml)/0.02% NaN_3_ (0.02% [w/v]). Half of the cell suspension was then mixed with 1 ml of 10% trichloroacetic acid (TCA). TCA precipitates were collected onto a 25 mm glass microfiber filter paper (Whatman GF/C), and the [^35^S] radioacitivity was measured in a scintillation counter. The TCA-precipitable counts were normalized against the counts of the other halves of the samples that were not precipitated by TCA. Each experiment was done in triplicates.

### Bicistronic dual reporter assay

The bicistronic dual reporter construct was modified from psiCHECK2 (Promega), which harbors a Renilla luciferase ORF and a firefly luciferase ORF under the control of SV40 and HSV-TK promoters, respectively. The psiCHECK2 was first digested with PmeI and ApaI (30 bp downstream of the firefly luciferase gene) for replacing the region between the two luciferase genes with a DNA fragment containing HCV-IRES (genotype-1). This HCV-IRES-containing fragment, which was obtained from PCR, has the following features (from 5′ to 3′): PmeI site, HCV-IRES sequence (i.e., HCV 5′UTR and coding sequence for the first 12 amino acids of the core protein), an alanine codon, coding sequence for the first 10 amino acids of firefly luciferase, and ApaI site. Transduction of Huh7.5 cells was done as described above, except that cells were plated in 96-well plates (5,000 cells per well). Transfection of the bicistronic dual reporter plasmid DNA (100 ng per well) was performed at post-transduction day 3 using Trans-IT LT1 transfection reagent (Mirus Bio). Transfected cells were harvested 20 h after transfection for luciferase activity assay using Dual-Glo Luciferase Assay System (Promega).

### Meta-analysis of a functional genomic screen

Data of the published functional genomic screen [19] were retrieved in Excel format and key-word sorted into the following categories: small subunit ribosomal proteins, large subunit ribosomal proteins, translation initiation factors, cyclin, cyclin-dependent kinases, and cell division cycle-related genes (see Dataset S1). The genomic screen employed a bicistronic subgenomic HCV replicon, in which the first cistron encodes a fused ORF containing a firefly-luciferase gene and a neomycin-resistance gene. Firefly luciferase activities therefore reflect the abundance of the bicistronic subgenomic HCV replicon. The retrieved Z score is the number of standard deviations of the experimental luciferase activity above the median plate value. Negative Z scores indicate inhibition of HCV replication. The distribution of the Z scores in each category was then analyzed and displayed as box plot using the NCSS 2007 software, with each box representing the interquartile range within which the middle 50% of the ranked data were found.

## Supporting Information

Dataset S1 **Meta-analysis Excel file supplementary to** **Figure 6** **.** (Sheet 1: Tai et al. 2009) The published complete screen result of the functional genomic siRNA screen. We used this data for meta-analysis and evaluation of the effects of knockdowns of specific gene categories individually. The overall effect on HCV level of each siRNA treatment is indicated as Mean Z score luciferase; on cell survival, Mean Z score CellTiterGlo. (Sheet 2: ribosomal protein s) Over 80% of siRNAs targeting small subunit ribosomal proteins significantly reduced Mean Z score luciferase, meanwhile their effects on cell survival are negligible. (Sheet 3: ribosomal protein l) For large subunit ribosomal protein, less than 10% of siRNAs show significant inhibitory effect on HCV. (Sheet 4: EIF1) (Sheet 5: EIF2)(Sheet 6: EIF3)(Sheet 7: EIF4)(Sheet 8: EIF4)(Sheet 9: EIF4)(Sheet 10: EIF5)(Sheet 11: Cyclin)(Sheet 12: CDK)(Sheet 12: Cell division cycle)(Sheet 12: Known factors) For genes encoding subunits of eukaryotic translation factor 1–5, cyclin, CDK, CDC or other factors known to be involved in HCV cycle, less than 25% of siRNAs targeting the aforementioned categories show significant inhibitory effect on HCV. Such contrast highlights the unique role of small subunit ribosomal protein in HCV replication.
(XLS)

Figure S1 **Individual knockdowns of** ***RPS6*** **and other** ***RPS*** **genes result in cytostatic effects.** (A) The effect of *RPS6* silencing on the survival rate and luciferase activity of the tricistronic replicon cell in the loss-of-function screen. Error bars represent SD of averages of two independent experiments. *lacZ* shRNA was used as a negative control. (B) The effects of silencing *RPS* genes on cell proliferation curve of Huh7.5 cell. Cells were transduced with shRNAs targeting individual *RPS* genes, and then were fixed at different time points for further cell number count using microscopic image analysis software. Fluorescent stained nuclei image were shot and counted by Cellomics ArrayScan VTI HCS Reader. Cell number of each treatment presented here is the sum of cell number count under 10 different fields of microscopic view.
(TIF)

Figure S2 **The relative ratio of 40S/60S ribosomal subunits is significantly reduced in** ***RPS20*** **knockdown cells.** (A)(B) Huh7.5 cells transduced with *RPS20* shRNA vector or *lacZ* were harvested at post-transduction day 5. Polysome profile analysis of *RPS20* knockdown cells in the presence of EDTA. (C) Quantitative analysis of each peak area is shown in the lower panel. Error bars, SD of independent replicates. Specific integration area in the *lacZ* shRNA-transduced cells is set as 100%. The experiment was similar to Figure 5B.
(TIF)

Figure S3 **Defining integration areas of each peak in quantification of polysome profiles.** The boundary lines are indicated in gray color. (A)(B) For Figure S2.
(TIF)

Figure S4 **Defining integration areas of each peak in quantification of polysome profiles.** The boundary lines are indicated in gray color. (A) For Figure 3A; (B) for Figure 3B; (C) for Figure 5B.
(TIF)

Protocol S1 **Production of VSV-G-pseudotyped lentivirus expressing shRNA.**
(PDF)

Protocol S2 **Determination of the relative titer of VSV-G-pseudotyped lenvirus.**
(PDF)

Table S1 **Gene category distribution of human kinase & phosphatase subset.** In the shRNA subset used in our screen experiment, 80% of the shRNA clones target kinase and/or phosphatase genes, whereas the remaining 20% target genes of other various categories. *RPS6* is the only ribosomal protein gene included in this subset target gene, because RPS6 is the substrate of a kinase, namely, RPS6K (ribosomal protein S6 kinase).
(PDF)

Table S2 **shRNA information.** Basic information of the shRNAs used in the follow-up study, including target sequence and target gene NCBI number etc. The *lacZ* shRNAs are used as negative controls. All the shRNAs targeting ribosomal proteins exhibit good knockdown efficiencies as confirmed by qRT-PCR.
(PDF)

Table S3 **Primers and probes used in qRT-PCR.** The pairs of primer and probe used in our study were designed and chosen based on the information provided by Universal Probe Library Design Center.
(PDF)
