# Supplementary figures and images for: Attenuation of 40S Ribosomal Subunit Abundance Differentially Affects Host and HCV Translation and Suppresses HCV Replication
Source: PLoS Pathog. 2012 Jun 28;8(6):e1002766. doi: 10.1371/journal.ppat.1002766 (PMC3394201; doi:10.1371/journal.ppat.1002766)

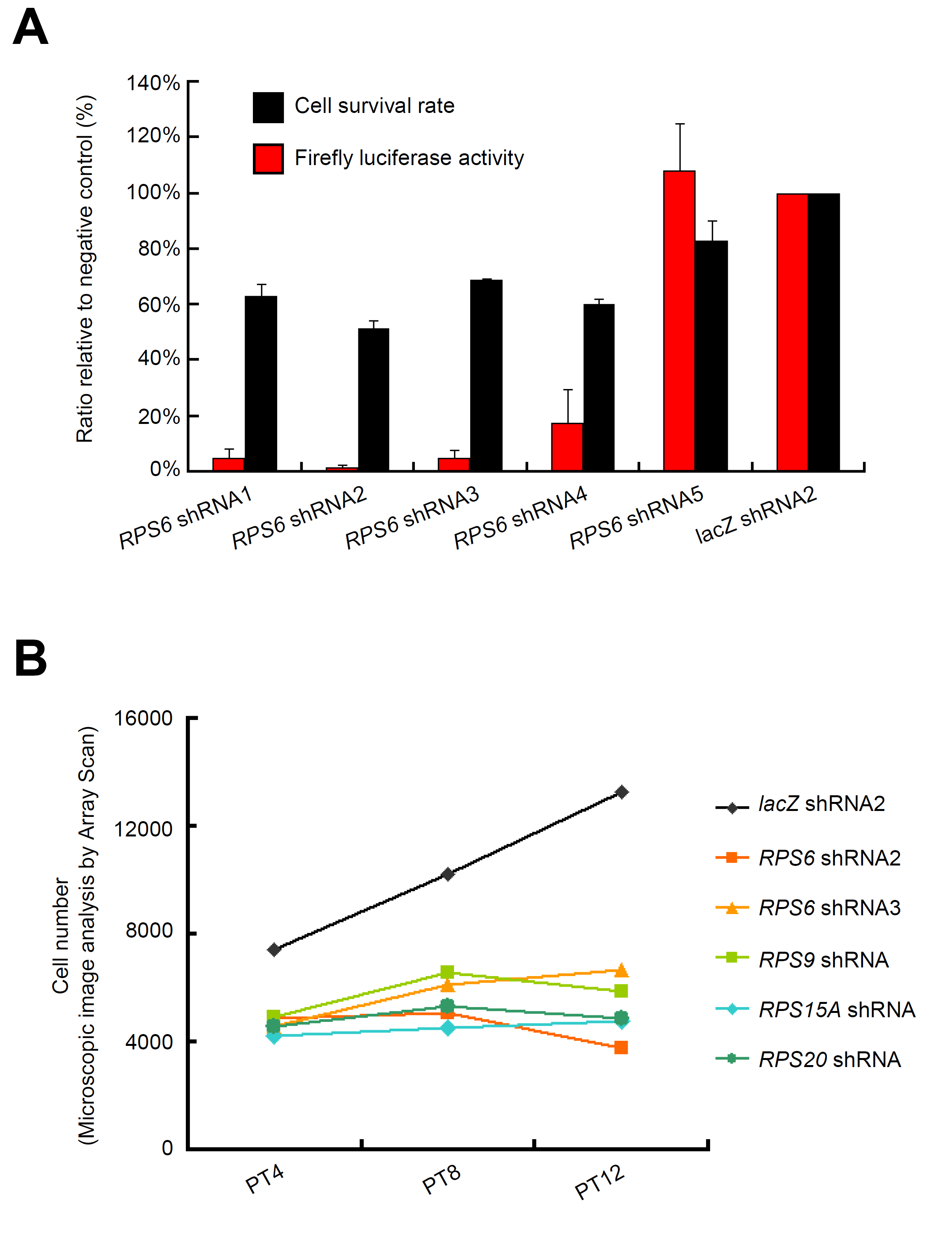

Supplement: Figure S1 — Individual knockdowns of RPS6 and other RPS genes result in cytostatic effects. (A) The effect of RPS6 silencing on the survival rate and luciferase activity of the tricistronic replicon cell in the loss-of-function screen. Error bars represent SD of averages of two independent experiments. lacZ shRNA was used as a negative control. (B) The effects of silencing RPS genes on cell proliferation curve of Huh7.5 cell. Cells were transduced with shRNAs targeting individual RPS genes, and then were fixed at different time points for further cell number count using microscopic image analysis software. Fluorescent stained nuclei image were shot and counted by Cellomics ArrayScan VTI HCS Reader. Cell number of each treatment presented here is the sum of cell number count under 10 different fields of microscopic view. (TIF) [file ppat.1002766.s002.tif]

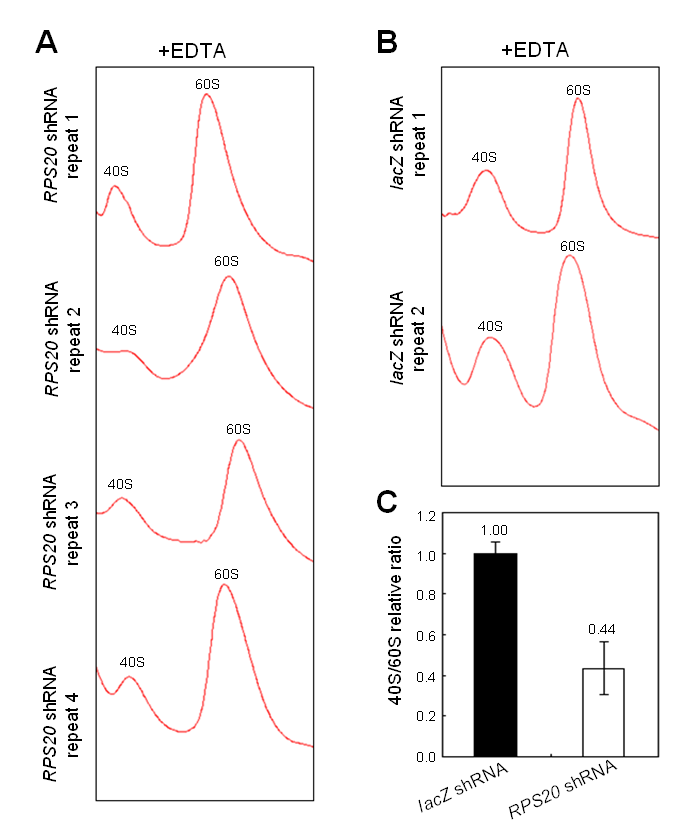

Supplement: Figure S2 — The relative ratio of 40S/60S ribosomal subunits is significantly reduced in RPS20 knockdown cells. (A)(B) Huh7.5 cells transduced with RPS20 shRNA vector or lacZ were harvested at post-transduction day 5. Polysome profile analysis of RPS20 knockdown cells in the presence of EDTA. (C) Quantitative analysis of each peak area is shown in the lower panel. Error bars, SD of independent replicates. Specific integration area in the lacZ shRNA-transduced cells is set as 100%. The experiment was similar to Figure 5B. (TIF) [file ppat.1002766.s003.tif]

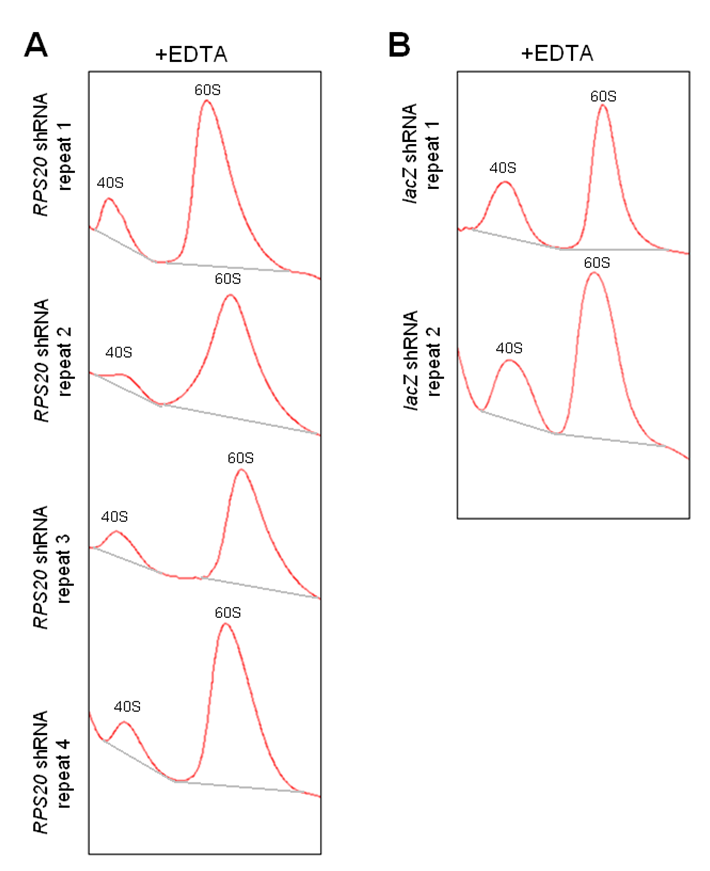

Supplement: Figure S3 — Defining integration areas of each peak in quantification of polysome profiles. The boundary lines are indicated in gray color. (A)(B) For Figure S2. (TIF) [file ppat.1002766.s004.tif]

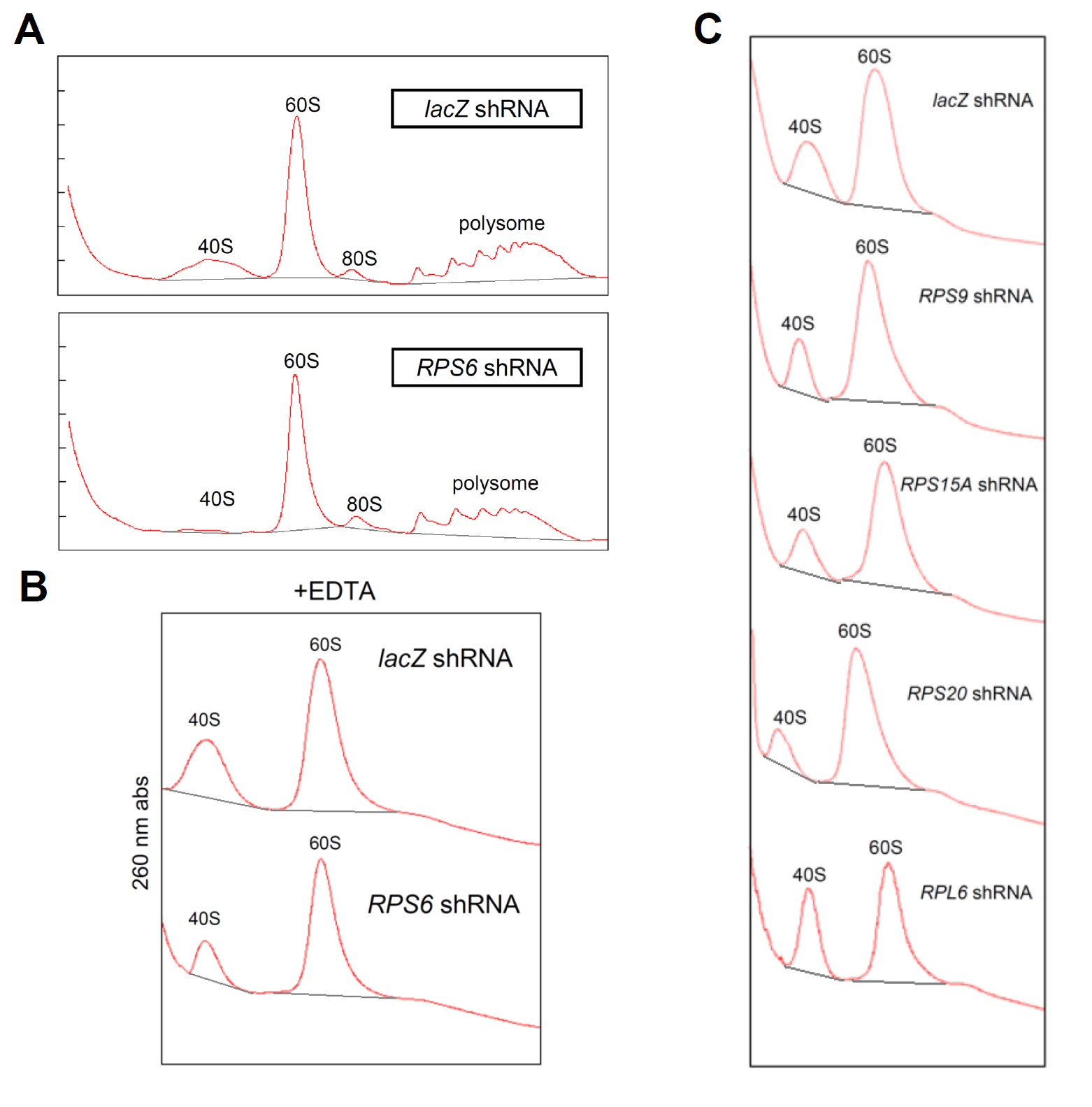

Supplement: Figure S4 — Defining integration areas of each peak in quantification of polysome profiles. The boundary lines are indicated in gray color. (A) For Figure 3A; (B) for Figure 3B; (C) for Figure 5B. (TIF) [file ppat.1002766.s005.tif]
