# Supplementary material for: Attenuation of 40S Ribosomal Subunit Abundance Differentially Affects Host and HCV Translation and Suppresses HCV Replication
Source: PLoS Pathog. 2012 Jun 28;8(6):e1002766. doi: 10.1371/journal.ppat.1002766 (PMC3394201; doi:10.1371/journal.ppat.1002766)
Supplement: Protocol S1 — Production of VSV-G-pseudotyped lentivirus expressing shRNA. (PDF) [file ppat.1002766.s006.pdf]

1/18/07

Section II: Lentiviral Production

Introduction:

This section contains protocols for the production of lentivirus stocks from hairpin-pLKO.1 plasmids in 6 cm plates and in high-throughput format (96-well plates).

Lentiviral production consists of the following steps:

Day 0	Seed 293T packaging cells
Day 1 (pm)	Transfect packaging cells with 3 lentivirus plasmids<br>(hairpin-pLKO.1 vector, packaging plasmid, envelope plasmid)
Day 2 (am)	*18 hours post-transfection*: Remove media; replace with fresh high-BSA or high-serum media
Day 3 (am)	*24 hours after media change*: Harvest virus; replace with fresh high-BSA or high-serum media
Day 4 (am)	*24 hours after harvest 1:* Harvest virus; discard packaging cells

These procedures should be carried out in accordance with biosafety requirements of the host institution

Part 1: Lentiviral Production in 6 cm plates

I. Materials

Transfection-quality plasmid DNA for:
- hairpin-pLKO.1 vector (TRC library plasmid – see Section I)
- 2nd generation packaging plasmid containing *gag*, *pol* and *rev* genes (e.g. pCMV-dR8.91 or pCMV-dR8.74psPAX2)*
- envelope plasmid (e.g. VSV-G expressing plasmid, pMD2.G)*

  ** recommended: use endotoxin-free plasmid isolation kits (Qiagen)*

TransIT-LT1 transfection reagent (Mirus Bio, MIR 2300/5/6)
  alternative: FuGENE 6 (Roche, #1 814 443 or #1 988 387)
OPTI-MEM serum-free media (Invitrogen, #31985-070)
293T packaging cells (recommended: passage number < 10)
Cell seeding media: Low-antibiotic 293T growth media (DMEM + 10% iFBS + 0.1x Pen/Strep)
  500 mL DMEM (Dulbecco's Modification of Eagle's Medium; e.g. Mediatech #10-013-CV)
  50 mL iFBS (heat-inactivated Fetal Bovine Serum; e.g. HyClone #SH30071.03)
  0.5 mL 100x Pen/Strep (10,000 IU/mL penicillin, 10,000 µg/mL streptomycin; e.g. Mediatech #30-002-CI)
Viral harvest media: High-BSA 293T growth media (DMEM + 10% iFBS + 1.1g/100mL BSA + 1x Pen/Strep)
  500 mL DMEM (Dulbecco's Modification of Eagle's Medium; e.g. Mediatech #10-013-CV)
  50 mL iFBS (heat-inactivated Fetal Bovine Serum; e.g. HyClone # SH30071.03)
  32 mL 20g/100mL BSA stock (microbiology-grade Bovine Serum Albumin; VWR #14230-738)
  5 mL 100x Pen/Strep (10,000 IU/mL penicillin, 10,000 µg/mL streptomycin; e.g. Mediatech #30-002-CI)
alternative viral harvest media: High-serum 293T growth media (DMEM + 30% iFBS + 1x Pen/Strep)
  500 mL DMEM (Dulbecco's Modification of Eagle's Medium; e.g. Mediatech #10-013-CV)
  200 mL iFBS (heat-inactivated Fetal Bovine Serum; e.g. HyClone # SH30071.03)
  5 mL 100x Pen/Strep (10,000 IU/mL penicillin, 10,000 µg/mL streptomycin; e.g. Mediatech #30-002-CI)
6 cm tissue culture plates
Polypropylene storage tubes

II. Instructions

1. Seed 293T packaging cells at 1.3-1.5x10^5 cells/mL (6 mL per plate) in low-antibiotic growth media (DMEM + 10% iFBS + 0.1x Pen/Strep) in 6 cm tissue culture plates.

2. Incubate cells for 24 hours (37 °C, 5% CO_2), or until the following afternoon. After ~24 hours, the cells should be ~70% confluent.

3. Transfect packaging cells:
   a. Prepare a mixture of the 3 transfection plasmids:

Reagent	per 6 cm plate
packaging plasmid (e.g. pCMV-dR8.91 or pCMV-R8.74psPAX2)	900 ng
envelope plasmid (e.g. VSV-G/pMD2.G)	100 ng
hairpin-pLKO.1 vector	1 µg
OPTI-MEM to total volume	10 to 30 µL*

   * The volume of OPTI-MEM per well can be adjusted for optimal handling.

   b. Dilute TransIT-LT1 transfection reagent in OPTI-MEM. Add the TransIT-LT1 reagent dropwise and mix by swirling the tip or gently flicking the tube (do not mix by pipetting or vortexing). Incubate 5 minutes at room temperature.

Reagent	per 6 cm plate
TransIT-LT1	6 µL
OPTI-MEM to total volume	90 µL

   c. Add the 3 plasmid mix dropwise to the diluted TransIT-LT1 reagent and mix by swirling the tip or gently flicking the tube.

   d. Incubate the transfection mix for 20 - 30 minutes at room temperature.

   e. Carefully transfer the transfection mix to the packaging cells (in low-antibiotic growth media). The packaging cells can be sensitive to perturbation - take care not to dislodge the cells from the plate. The total volume of transfection mix should be 100 to 125 µL per plate.

4. Incubate cells for 18 hours (37 °C, 5% CO_2), or until the following morning.

5. Change media to remove the transfection reagent and replace with 6 mL high-BSA growth media or high serum growth media for viral harvests.

6. Incubate cells for 24 hours (37 °C, 5% CO_2).

7. Harvest media containing lentivirus at ~40 hours post-transfection. Transfer media to a polypropylene storage tube. Replace with 6 mL high-BSA growth media or high serum growth media for viral harvests.

8. Repeat viral harvesting every 12-24 hours and replace with 6 mL high-BSA growth media or high serum growth media for viral harvests. Viral titer tends to decrease in later harvests; we typically collect a total of 2-3 time points. After the final harvest, discard the packaging cells. The viral harvests may be pooled as desired.

9. Spin the media containing virus at 1250 rpm for 5 minutes to pellet any packaging cells that were collected during harvesting. Transfer the supernatant to a sterile polypropylene storage tube.

10. Virus may be stored at 4 °C for short periods (hours to days), but should be frozen at -20 °C or -80 °C for long-term storage. To reduce the number of freeze/thaw cycles, aliquot large-scale virus preps to smaller storage tubes prior to long-term storage.

The RNAi Consortium

Part 2: High-Throughput Lentiviral Production (96 well plates)

I. Materials

Transfection-quality plasmid DNA for:
- hairpin-pLKO.1 vector (TRC library plasmid – see Section I)
- 2nd generation packaging plasmid containing *gag*, *pol* and *rev* genes (e.g. pCMV-dR8.91 or pCMV-dR8.74psPAX2)
- envelope plasmid (e.g. VSV-G expressing plasmid, pMD2.G)

TransIT-LT1 transfection reagent (Mirus Bio, MIR 2300/5/6)
alternative: FuGENE 6 (Roche, #1 814 443 or #1 988 387)
OPTI-MEM serum-free media (Invitrogen, #31985-070)
293T packaging cells (recommended: passage number < 10)
Cell seeding media: Low-antibiotic 293T growth media (DMEM + 10% iFBS + 0.1x Pen/Strep)
500 mL DMEM (Dulbecco's Modification of Eagle's Medium; e.g. Mediatech #10-013-CV)
50 mL iFBS (heat-inactivated Fetal Bovine Serum; e.g. HyClone #SH30071.03)
0.5 mL 100x Pen/Strep (10,000 IU/mL penicillin, 10,000 μg/mL streptomycin; e.g. Mediatech #30-002-CI)
Viral harvest media: High-BSA 293T growth media (DMEM + 10% iFBS + 1.1g/100mL BSA + 1x Pen/Strep)
500 mL DMEM (Dulbecco's Modification of Eagle's Medium; e.g. Mediatech #10-013-CV)
50 mL iFBS (heat-inactivated Fetal Bovine Serum; e.g. HyClone # SH30071.03)
32 mL 20g/100mL BSA stock (microbiology-grade Bovine Serum Albumin; VWR #14230-738)
5 mL 100x Pen/Strep (10,000 IU/mL penicillin, 10,000 μg/mL streptomycin; e.g. Mediatech #30-002-CI)
alternative viral harvest media: High-serum 293T growth media (DMEM + 30% iFBS + 1x Pen/Strep)
500 mL DMEM (Dulbecco's Modification of Eagle's Medium; e.g. Mediatech #10-013-CV)
200 mL iFBS (heat-inactivated Fetal Bovine Serum; e.g. HyClone # SH30071.03)
5 mL 100x Pen/Strep (10,000 IU/mL penicillin, 10,000 μg/mL streptomycin; e.g. Mediatech #30-002-CI)
96-well tissue culture plates (e.g. Corning/Costar #3628)
96-well polypropylene storage plates (e.g. Corning/Costar #3357)

II. Instructions

1. Seed 293T packaging cells at $2.2x10^5$ cell/mL (100 μL per well) in low-antibiotic growth media (DMEM + 10% iFBS + 0.1x Pen/Strep) in 96-well tissue culture plates. Allow seeded plates to sit undisturbed at room temperature for at least 1 hour before transferring to a tissue culture incubator overnight.
Note: allowing cells to settle at room temperature can reduce well-to-well variability and edge effects in microtiter plates.

2. Incubate cells for 24 hours (37 °C, 5% CO_2), or until the following afternoon. After ~24 hours, the cells should be ~70% confluent.

3. Transfect packaging cells:

   a. Prepare a mixture of the packaging and VSV-G envelope plasmids:

Reagent	per well*	per 96-well plate*
packaging plasmid (e.g. pCMV-dR8.91 or pCMV-dR8.74psPAX2)	100 ng	10 μg
envelope plasmid (e.g. VSV-G/pMD2.G)	10 ng	1 μg
OPTI-MEM to total volume	10 μL**	0.4 to 1 mL

   * Volumes do not account for excess dead volume required for multichannel pipette reservoirs or liquid handling robots.
   ** The volume of OPTI-MEM per well can be adjusted for optimal handling and automated setup.

   b. Dispense the packaging plasmid mix into a sterile 96-well polypropylene storage plate (10 μL per well). This plate will contain the transfection mix.

c. Dispense 100 ng hairpin-pLKO.1 plasmid into each well of the transfection mix plate. For 96-well transfections, it is convenient to normalize the concentration of hairpin-pLKO.1 plasmids to 20 ng/µL and dispense 5 µL per well.

d. Dilute TransIT-LT1 transfection reagent in OPTI-MEM. Add the TransIT-LT1 reagent dropwise and mix by swirling the tip or gently flicking the tube (do not mix by pipetting or vortexing). Incubate 5 minutes at room temperature.

Reagent	per well*	per 96-well plate*
TransIT-LT1	0.6 µL	60 µL
OPTI-MEM to total volume	10 µL**	1 to 1.5 mL

* Volumes do not account for excess dead volume required for multichannel pipette reservoirs or liquid handling robots.
** The volume of OPTI-MEM per well can be adjusted for optimal handling and automated setup.

e. Dispense the TransIT-LT1 mix to the transfection mix plate (10 µL per well) and mix gently by pipetting.

f. Incubate the transfection plate for 30 minutes at room temperature.

g. Carefully transfer the transfection mix to the packaging cells (in low-antibiotic growth media). The packaging cells can be sensitive to perturbation - take care not to dislodge the cells from the plate.

4. Incubate cells for 18 hours (37 °C, 5% CO_2), or until the following morning.

5. Change media to remove the transfection reagent and replace with 170 µL high-BSA growth media or high serum growth media for viral harvests.
   Note: Lentivirus will start to appear in the media supernatant ~22 hours post-transfection.

6. Incubate cells for 24 hours (37 °C, 5% CO_2), or until the following morning.

7. Harvest 150 µL media containing lentivirus and transfer to a 96-well polypropylene storage plate. Replace with 170 µL high-BSA growth media or high serum growth media for viral harvests.
   Note: The first harvest may be stored at 4 °C for 24 hours if the harvests will be pooled.

8. Incubate cells for 24 hours (37 °C, 5% CO_2), or until the following morning.

9. Harvest 150^+ µL media containing lentivirus and transfer to a 96-well polypropylene storage plate. Discard the packaging cells.

10. If desired, pool viral harvests and/or rearray to 96-well or 384-well plates. Virus may be stored at 4 °C for short periods (hours to days), but should be frozen at -20 °C or -80 °C for long term storage.

Version Notes:

1/18/07

High-BSA growth media for viral harvests: We have found that viral harvest growth media containing 10% serum + 1.1g/100mL supplemental BSA is equivalent to viral harvest media containing 30% serum – both produce viral stocks with similar high titer. The BSA-supplemented media is more cost effective, easier to mix in standard 500mL media bottles, and may be preferred when transfecting cells that are sensitive to serum.

Packaging plasmid: the pCMV-dR8.91and pCMV-dR8.74psPAX2 packaging plasmids are equivalent; both produce equivalent high-titer viral stocks. pCMV-dR8.74psPAX2 (“psPax2”) and the envelope plasmid pMD2.G are available from Addgene (www.addgene.org): psPax2 = plasmid #12260, pMD2.G plasmid #12259.

4/10/06

Transfection reagent: TransIT-LT1 (MirusBio) has the same performance as FuGene 6 (Roche) in our comparison tests. Either transfection reagent may be used for virus production. As of this version date, TransIT-LT1 has a lower list price.

Harvest volume and timeline: In previous HT protocols, we recommended 3 media harvests (100 µL each) at ~36, ~48, and ~60 hours post-transfection. We recover the equivalent (or higher) virus yield with 2 media harvests (150 µL each) at ~36 and ~60 hours post-transfection.

High-serum growth media: We have found that increasing the amount of serum to 30% in the virus production media improves virus yield by ~2-fold.
