# Supplementary material for: Attenuation of 40S Ribosomal Subunit Abundance Differentially Affects Host and HCV Translation and Suppresses HCV Replication
Source: PLoS Pathog. 2012 Jun 28;8(6):e1002766. doi: 10.1371/journal.ppat.1002766 (PMC3394201; doi:10.1371/journal.ppat.1002766)
Supplement: Protocol S2 — Determination of the relative titer of VSV-G-pseudotyped lenvirus. (PDF) [file ppat.1002766.s007.pdf]

Relative Viral Titering Using Cell Viability Assay (RIU Method) (Modified from TRC)

Introduction:

This section describes the protocol for a relative titering method for VSV-G-peudotyped lentivirus, based on selection for transduced cells and cell viability assay to quantify survival. According to the curve of cell viability versus viral dose, the relative titer would be estimated.

The relative titering assay consists of the following steps:

Day 1	Seed cells
Day 2	① Prepare virus dilutions<br>② Add diluted virus to cells in media containing polybrene<br>③ Centrifuge cells to promote infection<br>④ (Option!) Remove media and replace with fresh media if cells sensitive to polybrene
Day 3	*24 hours after virus addition:* Remove media; replace with media containing puromycin
Day 5	*48 hours after puromycin addition:* Remove media; replace with media containing 10% MTS. After 40-50 minutes incubation, record the absorbance at 490 nm using plate reader.

All lentiviral procedures should be carried out in accordance with biosafety requirements of the host institution.

I. Materials

96-well transparent tissue culture plate (Corning/Costar #3599)
A549 cell (do not use cells that have been cultured consecutively for more than 15 passages)
A549 growth media: F12K medium +10% FBS + 1x Pen/Strep.
Standard virus produced by RNAi Core (shLuc#221; C6-4-1)
Phenol red-free DMEM
96-well plate (Corning/Costar #3357; polypropylene)
Polybrene (Hexadimethrine bromide; Sigma H 9268, 8 mg/ml stock)
Puromycin Dihydrochloride (Sigma #P8833)
MTS kit (Promega G3581 or G1111; prepare stock solution according to manufacture's instruction)

Instruments

96-well plate reader (Absorbance OD=490 nm)

II. Procedures:

Day 1

1. Seed 100 µL per well of A549 cells (adjust A549 cells to a density of 60,000 cells/mL) in 96-well tissue culture plates. Allow seeded plates to sit undisturbed at BSC/ room temperature for 1 hour before transferring to a tissue culture incubator overnight (37°C, 5% CO_2).
Note: Allowing cells to settle at room temperature can reduce uneven distribution of cells.

Day 2

1. Remove 100 µl of growth medium and add 40 µl of growth medium containing 10 µg/ml of polybrene.

2. Prepare a series of diluted standard virus, with sufficient volumes for four replicate wells per dilution (5 µL will be used per transduction well).
   Example:

Make standard viruses as follows: (Use a new tip for each dilution)

Dilution number	*1*	*2*	*3*	*4*	*5*	*6*	*7*	*8*	*9*	*10*	*11*	*12*
Volume (µl) corresponding to stock virus in per 5µL transduction	2.5	2	1.5	1	0.8	0.6	0.4	0.3	0.2	0.1	0.05	0.03
**Media added** (µl)	**75**	**30**	**40**	**60**	**30**	**40**	**60**	**40**	**60**	**100**	**100**	**67**
**shLuc#221 virus stock or previous diluted virus added** (µl)	**75**	**120**	**120**	**120**	**120**	**120**	**120**	**120**	**120**	**100**	**100**	**100**
# Total volume (µl)	150	150	160	180	150	160	180	160	180	200	200	167
**Removed volume to next well/ dilution** (µl)	**120**	**120**	**120**	**120**	**120**	**120**	**120**	**120**	**100**	**100**	**100**	**0**
Remaining volume for transduction (µl)	30	30	40	60	30	40	60	40	80	100	100	167

3. Dilute virus being measured/ tested: add 5 µl of stock virus to 95 µl medium (20X dilution).
   Note: The final polybrene concentration is 8 µg/mL (following addition of virus).

4. Add 5 µl of both diluted standard virus and tested virus to each well of cells as follows:

Value in each well represents stock virus in per 5µL infection (shLuc#221)

	1	*2*	*3*	*4*	*5*	*6*	*7*	*8*	*9*	*10*	*11*	*12*
A												
B	2.5	2	1.5	1	0.8	0.6	0.4	0.3	0.2	0.1	0.05	0.03
C	2.5	2	1.5	1	0.8	0.6	0.4	0.3	0.2	0.1	0.05	0.03
D	2.5	2	1.5	1	0.8	0.6	0.4	0.3	0.2	0.1	0.05	0.03
E	2.5	2	1.5	1	0.8	0.6	0.4	0.3	0.2	0.1	0.05	0.03
F							Tested #1	Tested #2				
G							Tested #1	Tested #2				
H							Spike 2 µl of GFP virus	Spike 2 µl of GFP virus	0.2 No puro	0.1 No puro		

   Arrange tested viruses to remaining wells (duplicate or triplicate).

5. Spin plates at 1200Xg for 30 minutes at 37°C. Centrifugation can improve viral infection and decreases the length of exposure of cells to polybrene and virus.

6. Incubate cells overnight (37°C, 5% CO_2).

Day 3

1. Remove media and replace with 100 µL fresh growth media containing 2µg/mL puromycin.
   Note: ① Puromycin concentration may be adjusted for each batch of compound.
   ② Keep several uninfected wells without puromycin treatment.

2. Incubate cells for 48 hours (37°C, 5% CO_2).

Day 5

1. Remove media 48 hours post-puromycin addition and replace with 100 µL phenol red-free DMEM containing 10% MTS (V/V).

2. Incubate the plate for 40-50 minutes (37°C, 5% CO_2).

3. Record the absorbance at 490 nm using a 96-well plate reader.

4. Determine the relative viability by comparison with uninfected wells (setting as 100%). Generate the curve by plotting the survival rate versus viral dose. Define the liner range to determine the relative titer.

   Example:

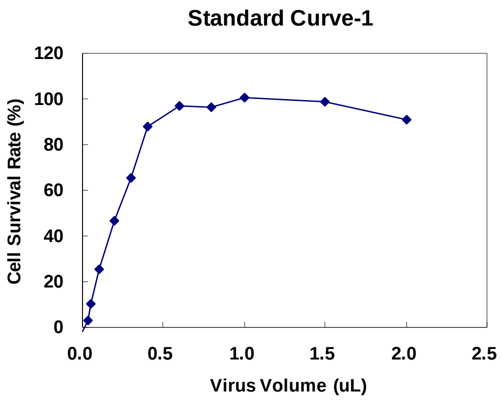

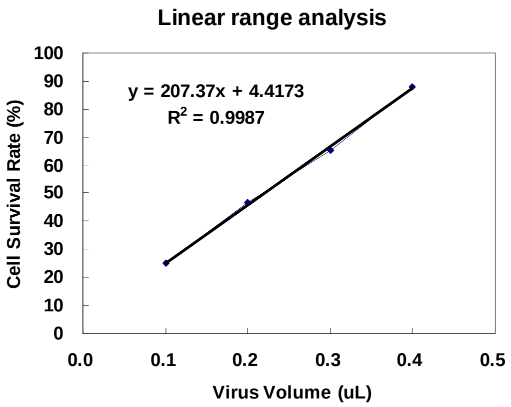

When A549 is infected by 0.4 µL original stock virus, the survival rate is about 88 %.
This is equivalent to 88% * 6000 (=5280) cells got infection.
The relative virus titer is: 5280 / 0.4 = 13200 R.I.U /µL ($1.3*10^7$ R.I.U./mL)
P.S.:R. I.U. stands for relative infection unit.
