# Supplementary material for: Attenuation of 40S Ribosomal Subunit Abundance Differentially Affects Host and HCV Translation and Suppresses HCV Replication
Source: PLoS Pathog. 2012 Jun 28;8(6):e1002766. doi: 10.1371/journal.ppat.1002766 (PMC3394201; doi:10.1371/journal.ppat.1002766)
Supplement: Table S1 — Gene category distribution of human kinase & phosphatase subset. In the shRNA subset used in our screen experiment, 80% of the shRNA clones target kinase and/or phosphatase genes, whereas the remaining 20% target genes of other various categories. RPS6 is the only ribosomal protein gene included in this subset target gene, because RPS6 is the substrate of a kinase, namely, RPS6K (ribosomal protein S6 kinase). (PDF) [file ppat.1002766.s008.pdf]

Table S1. Gene category distribution of Human kinase & phosphatase subset

Gene Category	Gene Number	Percentage
Kinase	737	59.63%
Phosphatase	209	16.91%
Kinase & Phosphatase	30	2.43%
subtotal	976	78.96%
Transcription factor	37	2.99%
Tumor suppressor	16	1.29%
Transcription factor &Tumor suppressor	15	1.21%
subtotal	68	5.50%
None	64	5.18%
Others	128	10.36%
subtotal	192	15.53%
Total	1236	100.00%

(1) RPS6 is phosphorylated by RPS6K, and therefore *RPS6* is included in this subset.

(2) *RPS6* is the only ribosomal protein gene included in this subset
