# Supplementary material for: Attenuation of 40S Ribosomal Subunit Abundance Differentially Affects Host and HCV Translation and Suppresses HCV Replication
Source: PLoS Pathog. 2012 Jun 28;8(6):e1002766. doi: 10.1371/journal.ppat.1002766 (PMC3394201; doi:10.1371/journal.ppat.1002766)
Supplement: Table S2 — shRNA information. Basic information of the shRNAs used in the follow-up study, including target sequence and target gene NCBI number etc. The lacZ shRNAs are used as negative controls. All the shRNAs targeting ribosomal proteins exhibit good knockdown efficiencies as confirmed by qRT-PCR. (PDF) [file ppat.1002766.s009.pdf]

Table S2. shRNA information

shRNA name	TRC Number	Target Gene	Reference sequence NM Number	shRNA target sequence
lacZ shRNA1	TRCN0000072229	*lacZ*	-	GCGATCGTAATCACCCGAGTG
lacZ shRNA2	TRCN0000072237	*lacZ*	-	CGCGCCTTTCGGCGGTGAAAT
RPS6 shRNA1	TRCN0000040079	*RPS6*	NM_001010	CCCAAGATTCAGCGTCTTGTT
RPS6 shRNA2	TRCN0000040080	*RPS6*	NM_001010	CGCAAACTTCGTACTTTCTAT
RPS6 shRNA3	TRCN0000040081	*RPS6*	NM_001010	CCGCCAGTATGTTGTAAGAAA
RPS6 shRNA4	TRCN0000040082	*RPS6*	NM_001010	GCTGCAGAATATGCTAAACTT
RPS6 shRNA5	TRCN0000040078	*RPS6*	NM_001010	GCCCTTAAATAAAGAAGGTAA
RPS9 shRNA	TRCN0000074794	*RPS9*	NM_001013	GCTGAAGCTGATCGGCGAGTA
RPS15A shRNA	TRCN0000074864	*RPS15A*	NM_001019	GCATGGTTACATTGGCGAATT
RPS20 shRNA	TRCN0000117625	*RPS20*	NM_001023	GATCGTTTCCAGATGAGAATT
RPL6 shRNA	TRCN0000004997	*RPL6*	NM_000970	CGGGTGGTTAAACTTCGCAAA
