# Supplementary material for: Attenuation of 40S Ribosomal Subunit Abundance Differentially Affects Host and HCV Translation and Suppresses HCV Replication
Source: PLoS Pathog. 2012 Jun 28;8(6):e1002766. doi: 10.1371/journal.ppat.1002766 (PMC3394201; doi:10.1371/journal.ppat.1002766)
Supplement: Table S3 — Primers and probes used in qRT-PCR. The pairs of primer and probe used in our study were designed and chosen based on the information provided by Universal Probe Library Design Center. (PDF) [file ppat.1002766.s010.pdf]

Table S3. Primers and probes used in qRT-PCR

Gene Name	probe number	Primer Name	Sequence
RPS6	#12	RPS6-NM001010-#12-L	AGGCGTTCAGCTGCTTCA
		RPS6-NM001010-#12-R	TACGAAGTTTGCGTTCATCG
HCV 5'UTR	#75	HCV 5'UTR-#75-L	CATGGCGTTAGTATGAGTGTCG
		HCV 5'UTR-#75-R	GGTTCCGCAGACCACTATG
PBGD	#25	PBGD-NM00190-#25-L	AGGATGGGCAACTGTACCTG
		PBGD-NM00190-#25-R	TCCTCAGGGCCATCTTCAT
PKR	#54	PKR-NM002759-#54-L	TGTTGGGATGGATTTGATTATG
		PKR-NM002759-#54-R	GAAAAGGCACTTAGTCTTTGACCT
AK3	#29	AK3-NM016282-#29-L	ATAGCTGGCTGTTGGATGGT
		AK3-NM016282-#29-R	CATTCAGGTTAATCACTGTGTCG
APOB	#79	APOB-NM000384-#79-L	CGGAAAATGGAGCCTAAAGA
		APOB-NM000384-#79-R	GGAGAAGCATCATCAAGGAAA
RPS9	#28	RPS9-NM001013-#28-L	CTGCTGACGCTTGATGAGAA
		RPS9-NM001013-#28-R	CAGCTTCATCTTGCCCTCA
RPS15A	#85	RPS15A-NM001019-#85-L	GGTGATCAGCCCCAGATTT
		RPS15A-NM001019-#85-R	CATGATGCCAGCTGAGGTT
RPS20	#10	RPS20-NM001023-#10-L	GAACAAGTCGGTCAGGAAGC
		RPS20-NM001023-#10-R	GATTTTACGTTGCGGCTTGT
Beta-actin	#64	Beta-actin-X00351-#64-L	CCAACCGCGAGAAGATGA
		Beta-actin-X00351-#64-R	CCAGAGGCGTACAGGGATAG
